# The Effects of Gender Trouble: An Integrative Theoretical Framework of the Perpetuation and Disruption of the Gender/Sex Binary

**DOI:** 10.1177/1745691620902442

**Published:** 2020-05-06

**Authors:** Thekla Morgenroth, Michelle K. Ryan

**Affiliations:** 1 Department of Psychology, University of Exeter; 2 Faculty of Economics and Business, University of Groningen

**Keywords:** gender binary, feminism, gender trouble, patriarchy

## Abstract

In the Western world, gender has traditionally been viewed as binary and as following directly from biological sex. This view is slowly changing among both experts and the general public, a change that has been met with strong opposition. In this article, we explore the psychological processes underlying these dynamics. Drawing on previous work on gender performativity as well as gender as a performance, we develop a psychological framework of the perpetuation and disruption of the gender/sex binary on a stage that facilitates and foregrounds binary gender/sex performance. Whenever character, costume, and script are not aligned the gender/sex binary is disrupted and gender trouble ensues. We integrate various strands of the psychological literature into this framework and explain the processes underlying these reactions. We propose that gender trouble can elicit threat—personal threat, group-based and identity threat, and system threat—which in turn leads to efforts to alleviate this threat through the reinforcement of the gender/sex binary. Our framework challenges the way psychologists have traditionally treated gender/sex in theory and empirical work and proposes new avenues and implications for future research.

Views of gender and sex are changing, both among experts and the general public (Hyde, Bigler, Joel, Tate, & van Anders, 2019; Schudson, Beischel, & van Anders, 2019). In the modern Western world, gender has traditionally been viewed as binary and oppositional (women vs. men) and as following directly from biological sex (female vs. male). These beliefs can be referred to as the gender/sex binary. In more recent years, however, views of gender and sex have become more fluid, as reflected in societal changes such as the growing visibility of, and support for, transgender and nonbinary individuals (e.g., Virginia electing the first trans woman as state legislator; Grierson, 2017), discussion and implementation of gender-inclusive language (e.g., gender-neutral pronouns such as “ze” and “they”; Boylan, 2018), and related changes to policy and practice (e.g., Germany’s top court legally recognizing a third sex; Eddy & Bennett, 2017).

At the same time, there has been stark opposition to these changes. Opponents argue that biological sex is binary and determines gender—and should therefore form the basis for policy and practice. It is noteworthy that these arguments are used by a range of groups that otherwise seem to have little in common, such as some religious groups who argue that more fluid views of gender threaten “family values” (Korolczuk & Graff, 2018) and some feminists who argue that they pose a threat to female voices and women’s safety (see Hines, 2019).

Although these changing views of gender/sex, as well as the societal changes and opposition that come with them, have received much attention in disciplines such as gender studies, sociology, and philosophy, these issues are largely absent from the psychological literature (for some notable exceptions, see Bem, 1995; Gustafsson Sendén, Bäck, & Lindqvist, 2015; Hyde et al., 2019; C. T. Nagoshi, Cloud, Lindley, Nagoshi, & Lothamer, 2019; see also Ansara & Hegarty, 2014; Morgenroth & Ryan, 2018). This absence seems somewhat surprising given the field’s interest in gender/sex more broadly—and in particular social psychology’s interest in inequality, intergroup relations, and social change. We argue that there is a large body of psychological research, particularly in social psychology, that is well placed to speak to these questions and has the potential to add to our understanding by providing insights into the psychological mechanisms that underlie the disruption and reinforcement of the gender/sex binary. At the same time, psychologists studying gender/sex would benefit from integrating ideas from disciplines that have substantially engaged with the reinforcement of the gender/sex binary into their understanding of sex and gender, as we have noted previously (see Morgenroth & Ryan, 2018). More specifically, we have argued that social psychology would benefit from an engagement with feminist philosopher Judith Butler’s concepts of *gender performativity* (i.e., that gender is created through its own performance) and *gender trouble* (i.e., ways to challenge the performative, reinforcing cycle of the gender/sex binary). Such a perspective is not only compatible with psychological theory and findings on gender/sex but also adds to an understanding of these theories and findings in that it changes the construct of gender/sex from a simple, binary, and stable variable that predicts psychological outcomes to a complex and dynamic construct that in itself is an *outcome* shaped by context and psychological processes. The unique complementarity between Butler’s ideas and psychological theories can help us understand the perpetuation and disruption of the gender/sex binary, as well as possible reactions individuals may have to these disruptions.

In this article, we propose an integrative framework of the perpetuation and disruption of the gender/sex binary. We draw on Butler’s work on gender performativity from her book *Gender Trouble* (Butler, 1990) as well as Goffman’s work on gender as a performance that suggests that gender/sex is something that is *done* in front of an audience rather than an inherent, biological quality (Goffman, 1959). In doing so, we integrate various strands of the extant psychological literature into this framework to facilitate an explanation of the psychological processes underlying these dynamics. This framework is novel in its integration of sociological, philosophical, and psychological theory, as well as its further integration of disparate strands of the psychological literature that speak to the same issue.

This article has four aims: (a) to develop a comprehensive psychological framework for understanding the nature of gendered performance, the contextual stage on which such a performance is set, and the role of the audience; (b) to integrate different strands of psychological literature within this framework to help explain reactions to challenges to the gender/sex binary; (c) to develop the basis for clear, testable research questions to stimulate future research in this area; and, perhaps most importantly; and (d) to challenge the way that psychologists treat gender/sex in theory and empirical work.

We first establish and justify the following assumptions of our model: (a) that gender is not binary, (b) that sex is not binary, (c) that gender does not follow from sex, (d) that the distinction between sex and gender is not always useful, and (e) that the gender/sex binary is harmful. We then propose a framework that outlines the inner workings of the gender/sex binary and the ways in which it can be disrupted. More specifically, we argue that binary views of gender/sex are created and reinforced through the performance of gender/sex in which there is an alignment between character (man vs. woman), costume (body and appearance), and script (gendered behavior, traits, and preferences); this performance is highlighted by a stage set up to facilitate performance in line with the gender/sex binary and obfuscate performance that does not fit binary notions of gender/sex. The audience observes and its members react to this performance.

After establishing our framework, we focus on the psychological processes involved in the reinforcement and disruption of the gender/sex binary, explaining when and why members of the audience may be motivated to uphold binary views and when they may not. More specifically, we propose that disruptions to the gender/sex binary can lead to different types of threat (personal threats, group-based and identity threats, and system threats), and, in turn, to efforts to reinforce the gender/sex binary. In the last section of this article, we highlight ways in which gender trouble can nevertheless be a catalyst to social change and discuss future research directions and implications arising from our framework.

## Assumptions and Terminology: Sex, Gender, and the Gender/Sex Binary

The gender/sex binary refers to the belief that both sex and gender are binary and that gender follows directly from sex (Butler, 1990; Hyde et al., 2019). In other words, individuals are either (a) born with two X chromosomes, a vagina, ovaries, and a uterus and will grow up to develop breasts, produce high levels of estrogen, produce ova, and have the ability to carry children or (b) born with an X and a Y chromosome, a penis and testicles, and will grow up to produce high levels of testosterone, produce sperm, and have the ability to father children. Those born with two X chromosomes will grow up to identify as, and fulfill the social role of, women, whereas those born with an X and a Y chromosome will grow up to identify as, and fulfill the social role of, men.

“Sex” in this context refers to the biological makeup of an individual (such as their chromosomes, sex characteristics, and hormones), whereas “gender” has been used to refer both to the cultural interpretation of sex (i.e., gender roles and stereotypes, what it means to be a woman or a man in a given society) and to gender identity (i.e., self-categorization into the groups “girls” and “boys” or “women” and “men,” respectively; American Psychological Association, 2018; Prince, 2005; Wood & Eagly, 2015). In line with this distinction, it has been argued that the term sex should be used when referring to differences between men and women that stem from biology or “nature,” whereas the term gender should be used when referring to differences that are produced by socialization or “nurture” (see Hyde et al., 2019).

The gender/sex binary dictates not only which genders exist but also how they are linked to sex. Many authors, including Butler (1990), go further and argue that the gender/sex binary also dictates sexual desire. Butler argues that within Western culture, sex, gender, and sexual orientation are closely interconnected in what she calls the “heterosexual matrix.” This matrix dictates that biological sex is binary (male vs. female) and forms the basis for binary gender (women and men) as well as (heterosexual) sexual attraction. In other words, such a worldview expects that babies will all be born as clearly male or female and that they will grow up to identify with the respective category and act accordingly—including being sexually attracted to the opposite sex. Similar views are expressed by psychologists, who see heterosexuality as the core part of gender roles, particularly for men (Herek, 1986).

However, although these views are deeply ingrained in our culture, they do not reflect reality. Neither sex nor gender is clearly binary and neither gender nor sexual desire necessarily follows from sex.

### Gender is not binary

A large body of evidence demonstrates that in terms of traits, abilities, interests, and behaviors, men and women do not clearly fall into two distinct categories (Hyde, 2005). Although there are indeed some average differences between men and women on these variables, the vast majority of these differences are small and show a large overlap between the groups. Indeed, even for differences that would be considered large by psychologists (*d* ≥ 0.80), the overlap between the two distributions is still 68.92% (Magnusson, 2014)—far from what biologists would consider dimorphic (i.e., two distinct, largely nonoverlapping categories; see Hyde et al., 2019). In line with this finding, research further shows that gender (examined in a range of ways, including sexual attitudes and behaviors, interpersonal orientation, and personality traits) is dimensional rather than taxonic (i.e., forming two distinct categories with groups of attributes such as aggression, mathematical ability, and short-term mating goals all clustering together; Carothers & Reis, 2013). This is further illustrated by the fact that individual women and men exhibit a mix of both “feminine” and “masculine” attributes and engage in both “feminine” and “masculine” behaviors, making the claim that women and men are psychologically distinct implausible (Hyde et al., 2019; Joel et al., 2015). This holds true even for behaviors for which large gender/sex differences have been found (e.g., pornography use, taking a bath)—they are exhibited by both women and men.

When “gender” is interpreted to mean “gender identity” in the sense of self-categorization, nonbinary individuals offer clear evidence against gender being binary. Nonbinary individuals are those who identify as neither exclusively male nor exclusively female. This can include, but is not limited to, identifying as gender fluid (not having a fixed gender), multigender (having more than one gender), or agender (having no gender; LGBT Foundation, 2017). Although most of the population does identify as women or men, approximately 1 in 250 people identified as nonbinary in a representative survey from the United Kingdom (Glen & Hurrell, 2012), and even among cisgender women and men (i.e., those whose gender identity matches their sex assigned at birth), 35% say that they feel, at least to some extent, like the other gender (Joel, Tarrasch, Berman, Mukamel, & Ziv, 2014). Thus, neither gender interpreted as the cultural interpretation of sex nor gender interpreted as gender identity is binary.

### Sex is not binary

There are some clear biological differences between females and males. Most individuals with two X chromosomes do indeed develop clearly female sex characteristics, and most individuals with an X and a Y chromosome develop clearly male sex characteristics. However, research from a range of disciplines such as biology, neuroscience, and neuroendocrinology challenge the idea that sex is binary and instead suggest that sex is a spectrum (Ainsworth, 2015; Fausto-Sterling, 1993, 2000; Hyde et al., 2019). In addition, as Mol (1985, 2015) points out, it is unclear which exact biological differences determine sex, and different biological subdisciplines such as anatomy, endocrinology, and genetics may offer different—and sometimes contradictory—definitions.

Biologists argue that the view that having a Y chromosome is equivalent to being male is overly simplistic because there are many cases for which this is not true (Ainsworth, 2015; Fausto-Sterling, 1993, 2000). For example, some individuals are born with a mixture of XY and XX cells or absorb XY cells during pregnancy. For others, chromosomes indicate one sex but gonads and other sex characteristics another. Yet for others, sex characteristics are ambiguous and neither clearly female nor clearly male (see Ainsworth, 2015). Thus, there are a number of different ways in which individuals can be considered intersex—that is, biologically neither clearly female nor clearly male. Exact numbers are heavily disputed—estimates range from 0.018% of the population (Sax, 2002) to 10% of the population (Arboleda, Sandberg, & Vilain, 2014)—but this very discussion illustrates that there is no easy or clear definition of sex and that, regardless of the definition, some individuals will fall outside of the categories female and male.

Findings from neuroscience similarly show no evidence for two distinct sexes (see Fine, 2010; Hyde et al., 2019). Although research on brain structures consistently finds some sex differences, these differences are not dimorphic. Instead, like gendered psychological attributes, the distributions overlap extensively, and the vast majority of brains are made up of a mix of female and male features that do not cluster together in a way that creates a clear female-to-male continuum. Such features include, for example, the connection between the left superior temporal gyrus and the left middle temporal gyrus, which is stronger in males than in females, and the gray-matter volume of the caudate nuclei, which is higher in females than in males (Joel et al., 2015). Moreover, sex differences that do exist depend on contextual factors (e.g., research on rodents suggests that these differences can be reversed under conditions of stress; Reich, Taylor, & McCarthy, 2009) and develop over time (Fine, 2010; Hyde et al., 2019), illustrating that they are by no means “hardwired.”

Findings from behavioral neuroendocrinology are similar and demonstrate that there is no hormonal evidence for two distinct sexes (Hyde et al., 2019). First, both “female” hormones (i.e., estrogens such as estradiol) and “male” hormones (i.e., androgens such as testosterone) are produced by both females and males, and, once more, the average circulating levels of these hormones are not dimorphic but instead overlap considerably between females and males. Indeed, average levels of estradiol, for example, do not differ between males and females (Liening, Stanton, Saini, & Schultheiss, 2010), and estradiol levels of nonpregnant females are more similar to those of males than those of pregnant females (Tulchinsky, Hobel, Yeager, & Marshall, 1972). The latter point further illustrates that these hormone levels are not fixed. They vary across the life span and depend on many contextual factors, including social and psychological factors such as sexual thoughts, relationship transitions, and power (see Fine, 2017; Hyde et al., 2019; van Anders, Steiger, & Goldey, 2015).

In summary, neither our anatomical sex characteristics nor our brains or hormones are clearly binary. Although most people can be classified as female or male on the basis of their chromosomes, gonads, and other sex characteristics, this is not the case for all individuals, and a clear classification on the basis of brain structures or hormones is not possible. Moreover, intersex individuals provide clear evidence against the claim that individuals can be divided into two groups on the basis of sex.

### Gender does not always follow from sex

We have argued thus far that neither gender nor sex is binary. We now tackle the third assumption of the gender/sex binary: that gender follows from sex. Trans and nonbinary individuals pose a clear challenge to this assumption, as their gender identity differs from their sex assigned at birth. Although “trans” is generally used as an umbrella term that includes binary and nonbinary identities (see Levitt, 2019), we use it to refer to individuals whose gender identity is the “opposite” of the sex they were assigned at birth; a trans man is thus a man who was assigned the sex female at birth, whereas a trans woman is a woman who was assigned the sex male at birth. We distinguish trans women and men from nonbinary individuals, that is, those who reject binary labels, as this distinction will become important at different points throughout this article. We use the terms trans and nonbinary to include both those who have medically transitioned (e.g., via gender confirmation surgery, hormone replacement therapy) and those who have not and may or may not desire to medically transition.

It is important to note that trans and nonbinary individuals are by no means a modern or Western phenomenon. Indeed, there is evidence for the existence of nonbinary and trans people throughout history and across a range of cultures (Herdt, 1993). In addition, recent research indicates that the gender identity of trans children develops early and that gender development is remarkably similar to that of cisgender children, for example, in terms of consistency of gender identity (Olson & Gülgöz, 2018). Trans girls exhibit patterns of gender development almost identical to that of cis girls—and very different from that of cis boys (i.e., the sex they were assigned at birth)—whereas the development of trans boys is almost identical to that of cis boys and different from that of cis girls, for example, in terms of gender-typical preferences. These patterns have been demonstrated using both explicit and implicit measures (Olson, Key, & Eaton, 2015) and are in line with research indicating that gender-minority individuals experience gender identity as reflecting a deep, innate, and immutable sense of self (Levitt, 2019).

This does not imply, however, that gender identity follows from sex—be it anatomical, neurological, or hormonal. Instead, research indicates that gender identity develops in response to the gender labels and roles available and known to individuals in a particular context or culture (Levitt, 2019). This also means that gender-identity labels can change over time, even if the internal sense of gender does not. For example, trans men may initially identify as “butch” lesbians before learning more about trans male identities that better fit their internal sense of self (Devor, 1997). In many cases, an individual’s internal sense of gender may fit with the sex assigned to them at birth and thus with one of the two most commonly available gender labels. At the same time, it is impossible to know whether this would still be the case if more gender labels were known and available to children from the start. Indeed, the fact that an increasing number of individuals—particularly children and young people—reject their sex assigned at birth (Flores, Herman, Gates, & Brown, 2016; Gender Identity Development Service, 2019; Higher Education Statistics Agency, 2018) as other options are becoming more visible and less stigmatized, especially among young people (see Rivas, 2015), indicates that assigned at birth is not the best fit for many individuals’ internal sense of gendered self.

In summary, the existence of trans and nonbinary people across time and culture, as well as evidence that transgender identities develop early and consistently, demonstrates that these identities are not a quirk of current times or our current culture but that gender does not always follow from sex. At the same time, cultural context does affect individuals’ specific gender labels, further illustrating that biology alone cannot explain gender identity.

### Is the distinction between gender and sex meaningful?

We noted above that the term *sex* is generally used to refer to biological differences between females and males, whereas *gender* is used to refer to cultural associations with the female and male sex (i.e., gender roles) or to self-categorization into the categories of women and men (i.e., gender identity). Several scholars have argued, however, that this distinction is neither straightforward nor always particularly useful (Butler, 1990; Fausto-Sterling, 2019; Hyde et al., 2019; van Anders, 2015; Yoder, 2003). This may seem to contradict the point we have just made—that gender does not follow from sex. However, these arguments do not suggest that gender is inherently linked to sex in the way the gender/sex binary dictates (i.e., that gender identity and gendered behavior are biologically determined). Rather, these scholars suggest that sex itself is a social construct or that sex is always also affected by gender, a point that is illustrated by the fact that sex is defined in different, at times contradictory, ways by different disciplines (see Mol, 2015).

Butler (1990), for example, rejects the idea that sex is natural and prediscursive, that is, that it exists before cultural interpretation. Although Butler does not deny that biological differences exist, she argues that it is only because of our culture and because of our binary views of gender/sex that we interpret these bodies as male and female and perceive them, for the most part, as falling into two clear and distinct categories. She further makes the point that sex comes with just as many prescriptive and proscriptive rules as gender, concluding that “perhaps this construct called ‘sex’ is as culturally constructed as gender; indeed, perhaps it was always already gender with the consequence that the distinction between sex and gender turns out to be no distinction at all” (p. 9).

Similar sentiments were recently voiced by Hyde and colleagues (2019), who advocate for the use of the term gender/sex to indicate that there is no clear distinction between what is biological and what is sociocultural, as these aspects influence one another (see also van Anders, 2013). We agree with this sentiment and thus generally use gender/sex throughout this article. This term does not imply that the terms gender and sex can be used interchangeably—indeed, there are cases in which it is important to distinguish between them (e.g., when distinguishing between sex assigned at birth and gender identity); instead, it highlights the fact that these terms are closely connected both in the sense that biology and socialization mutually influence one another and in the sense that both are culturally created constructs. Indeed, we will use the terms sex and gender in cases in which we truly refer to only one or the other (e.g., when talking about gender identity specifically) and when the distinction is meaningful or when using terms introduced by others (e.g., “gender trouble”). Although *gender binary* could be argued to also be an established term, we use the term *gender/sex binary* to emphasize that it dictates not only which genders exist and how they should behave but also which sexes exist and how they are linked to gender.

### The gender/sex binary is harmful

The gender/sex binary is not only based on incorrect assumptions but also has a plethora of negative consequences for those who disrupt this restrictive framework and for society more generally. Our view on this echoes Butler, who argues that oppositional and discrete genders/sexes are seen as an essential part of humanness and that those who fail to perform their gender/sex “correctly” are punished by society. This punishment is aimed toward a range of groups and individuals, including trans, nonbinary, and intersex people (Human Rights Campaign, 2018; Seelman, 2014), as well as members of the LGB community (DeSouza, Wesselmann, & Ispas, 2017; Dyar & London, 2018; Katz-Wise & Hyde, 2012), but also cis women and men who violate gender norms, such as stay-at-home fathers or female leaders (Moss-Racusin, Phelan, & Rudman, 2010; Rudman, Moss-Racusin, Phelan, & Nauts, 2012). Negative consequences for these individuals can include anything from economic and social penalties (Rudman, Moss-Racusin, Glick, & Phelan, 2012) to extreme violence and even death (Human Rights Campaign, 2018). For example, a meta-analysis found that 28% of lesbian, gay, and bisexual individuals reported having been a victim of physical assault simply because of their sexual orientation (Katz-Wise & Hyde, 2012).

However, the pernicious effects of the gender/sex binary go beyond the direct impact for those who disrupt it; it sustains a gendered system of power imbalance that oppresses women (and other marginalized groups) as a group and encourages harmful behaviors in men, reflected in high levels of suicide and incarceration (Federal Bureau of Prisons, 2019; National Institute of Mental Health, 2019). In other words, it supports the patriarchy (see Butler, 1990; Wilton et al., 2019). Butler (1990) argues that the gender/sex binary upholds a patriarchal system of compulsory heterosexuality in which women’s purpose is to serve men as partners in reproduction, as their mothers, and as their wives. It is noteworthy that these power structures are both generative (i.e., prescriptive) in that they create ideas of what gender/sex looks like, and they are prohibitive (i.e., proscriptive) in that they repress deviance from gendered norms. Although not linking these views to the gender/sex binary directly, the literature on ambivalent sexism supports this notion, exploring a widespread and system-justifying ideology in which women are portrayed as morally pure caregivers (Glick & Fiske, 2001). This literature also shows that women who conform to these norms are seen as worthy of protection, but women who aim to upset the status quo are harshly punished (Glick & Fiske, 2001; Viki & Abrams, 2002). Likewise, social sanctions that stem from the social-role theory (Eagly, 1987; Eagly & Wood, 2012) are one of the key regulatory mechanisms that perpetuate gender/sex stereotypes and roles. Butler’s work is a call to action to overthrow these structures by creating gender trouble by subverting and disrupting the status quo via the repeated refusal to engage in binary gender/sex performance.

### Summary of section

In this section we outlined and justified the assumptions on which our model is based and explained key terms such as gender/sex, gender/sex binary, trans, and nonbinary. We argued that neither gender nor sex is binary and that gender does not always follow from sex—and that, therefore, the gender binary does not accurately reflect reality. That said, many of the examples we gave to justify our argument apply only to a minority of people: Most people identify as women or men; most people can be classified as clearly female or male on the basis of their anatomy; and most people’s gender identity matches their sex assigned at birth. Nevertheless, there are likely very few people whose gender/sex completely aligns with the assumptions of the gender/sex binary. For example, few people behave in only feminine or only masculine ways or have exclusively “female” or “male” brains, and thus the gender/sex binary falls short of describing the gender-identity experiences of many individuals, including cisgender people. Perhaps more importantly, the gender/sex binary harms even those who mostly adhere to its prescriptions and proscriptions (e.g., cisgender, heterosexual men who sometimes engage in some stereotypically feminine behaviors) and, as it is a patriarchal system, even those who completely adhere to them (e.g., cisgender, heterosexual women and men). As such, although the gender/sex binary may have some benefits, such as structuring a chaotic and complex world (Fiske, 2010), it is, overall, exclusionary, harmful, inaccurate, and not particularly useful.

In the next section we describe our framework of the maintenance and disruption of the gender/sex binary before focusing on the psychological mechanisms underlying the reactions to gender trouble, that is, the disruption of the gender/sex binary.

## A Framework for Understanding the Perpetuation and Disruption of the Gender/Sex Binary

If neither sex nor gender is binary and gender does not follow from sex, why is the gender/sex binary so widespread and persistent? In this section, we put forward a framework that answers this question. Drawing primarily on work from Goffman (1959) and Butler (1990),^1^ we first describe how the gender/sex binary works and perpetuates itself through the performance of gender and how it can be disrupted. We then draw on psychological theory and evidence to explore the potential reactions to such disruptions, arguing that they can elicit threat and, in turn, efforts to reinforce the gender/sex binary.

### The inner workings of the gender/sex binary

We argue that the gender/sex binary is created and maintained through the socially regulated, binary performance of gender/sex (see Butler, 1990; see also Deaux & Major, 1987; Eagly & Wood, 2012). We propose that the maintenance of the gender/sex binary, via the performance of gender, can be broken down into four related but distinct facets: the *character* one plays, the *costume* one wears, the *scripts* one enacts to portray the character, and the *stage* on which this performance takes place. This performance of gender/sex takes place in front of the *audience* of others and the self.

With these metaphors we build on Goffman (1959), who introduced theater as a metaphor for the performance of gender. In his book *The Presentation of Self in Everyday Life*, Goffman argues that there is no “natural” or “true” inner gender. Rather, the gendered self arises only as a response to our performance of gender, which is in itself a form of impression management, and others’ reactions to this performance. In other words, although gender may *feel* like an innate identity, it emerges only in a social context that gives it meaning and importance, similar to other identities (e.g., national identities). Goffman uses the metaphor of theater and argues that we are all actors on a stage who play different roles in different contexts. It is important to note that these ideas are very much compatible with psychological theory and research. For example, the classic model of gender-related behavior proposed by Deaux and Major (1987) also focuses on how gender is *done* with respect to situational and contextual factors. Like Goffman, Deaux and Major propose that the performance of gender primarily takes place in social interactions.

We build on Goffman’s work but deviate both in terms of the conceptualization of some key aspects and, to reflect these differences, in terms of key terminology. We use the term character to refer to the social category (men or women) into which one is categorized—either by others or by oneself. We propose that character is generally constructed as essential (Haslam, Rothschild, & Ernst, 2000). Essentialism refers to the belief that group membership is biologically determined and stable (throughout history and throughout group members’ lives) and that group members share an underlying “essence” that makes them similar to one another and different from other groups (Rhodes & Gelman, 2009). Applied to gender/sex, it thus refers to the belief that gender/sex (i.e., the character one plays) is a stable construct based on biological sex.

The character is based on elements that are generally considered sex (i.e., sex assigned at birth) as well as those generally considered gender (i.e., gender identity), illustrating once more that it is not always useful or straightforward to separate these two terms. In most cases, categorization by others and categorization by oneself (i.e., gender identity) are aligned, but when they are not, gender trouble ensues, as we discuss in more detail below. In our conceptualization, the character one plays is based on an interplay of societal forces (e.g., the acceptability and availability of different gender labels in a culture or context) and an internal sense of self. This interplay becomes particularly apparent when the categorization by others and oneself do not match (i.e., for transgender and nonbinary people; see Levitt, 2019). However, like Goffman (1959), we argue that this internal sense of self is perceived as gendered (i.e., in line or in conflict with different gender labels) only because of the importance culture gives to gender/sex. Note that neither the societal forces that determine gender labels nor the internal sense of self is under individuals’ control. Thus, individuals—cis, trans, and nonbinary alike—have little choice in the character they play.

The costume is a central part of any performance. As outlined by Goffman (1959), even in the absence of behavior, the costume helps to communicate gender/sex to the audience. We define costume in broad terms, including aspects of the body itself (e.g., genitals and breasts, body and facial hair, and muscle mass—all of which align more with what is considered sex) as well as the presentation of the body (e.g., makeup and clothes—which align more with what is considered gender), all of which is informed by cultural gender norms (e.g., men are expected to be more muscular than women whereas women are seen as more likely to wear jewelry than men; see E. L. Haines, Deaux, & Lofaro, 2016). Although these two aspects might seem quite different from one another, like Butler (1990), we argue that it is impossible to distinguish between the body itself and the presentation of the body. For example, body hair can be argued to be part of the body itself, but its removal (or, indeed, its presence) is a choice guided by cultural, gendered norms. Thus, there is no “neutral” body—it always serves as a medium to communicate and perform gender/sex in one way or another.

Scripts are gendered behaviors that are also informed by gender norms and stereotypes and include a number of aspects such as gendered preferences (e.g., men liking cars, women liking dancing; Lippa, 2010) and gendered traits (e.g., women being emotional, men being competitive; see E. L. Haines et al., 2016). These preferences and traits are expressed through gendered behavior (e.g., watching a romantic movie, playing a sport), including verbal statements (e.g., “I like romantic movies”). In line with Butler’s (and others’) argument that heterosexuality is an integral part of our culture’s conceptualization of gender/sex, heterosexual desire and behaviors are particularly important parts of the gender/sex script (see also Herek, 1986). Thus, although most aspects of the script are more closely aligned with gender (particularly in the sense of gender norms), some aspects (e.g., sexual attraction) may be, at least in part, biologically based (see Bailey et al., 2016).

Costume and scripts are observable and are used by the performer to express their character (often in line with a deep-seated sense of gendered self) and by the audience to determine which character is being played (see Deaux & Lewis, 1984; Goffman, 1959). However, we would argue that the process also works the other way around. The audience uses the character (categorization as male or female) to make inferences about costume (e.g., genitals) and scripts (e.g., expectations of how someone is likely to behave). In other words, the audience use stereotypes associated with the character to predict and evaluate appearance and behavior (see Eagly & Wood, 2012; E. L. Haines et al., 2016).

It should be noted that costume and script can, and do, vary depending on intersecting identities such as race, ethnicity, sexual orientation, and class (e.g., Ghavami & Peplau, 2012; Kite & Deaux, 1987). Thus, how exactly gender/sex is performed—and is expected to be performed—may look quite different, for example, for a Black woman compared with a White woman, or for an Asian man compared with a White man (see Ghavami & Peplau, 2012). At the same time, in Western cultures in which White is seen as the default (see Purdie-Vaughns & Eibach, 2008; Sesko & Biernat, 2010), the performance of “White” (middle- or upper-class) femininity and masculinity is likely to be particularly valued and seen as the “best” way to perform one’s gender/sex (see Collins, 2004; Landrine, 1985), whereas femininity and masculinity among marginalized groups is used as a tool of oppression against them (see Donovan, 2011). In other words, marginalized groups may be expected to perform gender/sex differently but at the same time are devalued for it. However, intersecting identities can give rise to new, more empowering ways to perform gender, including scripts and costumes that disrupt the gender/sex binary. For example, some communities of gay men (i.e., leather men and bears) have redefined masculinity in a way that includes qualities such as vulnerability and nurturance (Graves, 2007; Mosher, Levitt, & Manley, 2006).

Characters, costumes, and scripts are part of the performance of gender/sex itself and all include descriptive, prescriptive, and proscriptive aspects. They describe what genders/sexes exist, what they look like, and how they behave. They also dictate what genders/sexes *ought* to exist, what they should and should not look like, and how they should and should not behave (see Eagly & Wood, 2012).

The stage, on the other hand, refers to the physical and cultural environment in which gender/sex is performed and is set up to enable and reinforce the performance of binary gender. It includes physical spaces (e.g., gender-segregated bathrooms) but also the broader backdrops of culture (e.g., gender roles), language (e.g., gendered pronouns), and laws (e.g., number of legally recognized sexes). Thus, although it is not directly part of gender/sex performance, it can highlight or obfuscate binary gender/sex performance. For example, addressing a mixed-gender group as “boys and girls” or “ladies and gentlemen” emphasizes the fact that there are two—and only two—genders/sexes with important differences, whereas using gender-neutral terms such as “y’all” or “folks” does not make this distinction.

The audience consists of others, as well as the self, who observe the performance and react to it. Thus, when gender/sex is performed correctly, the performance reinforces binary, oppositional ideas of gender/sex in the minds of the audience, including the self (see Eagly, 1987; Eagly & Wood, 2012). Our view on the audience deviates from Goffman’s conception in that we view the self as part of the audience, whereas he distinguishes between a “front stage,” where the audience is present and the performance of gender/sex is tailored toward them, and a “back stage,” where the audience is absent and the individual can act in a way that is tailored to their own wants and needs. We propose that there is no back stage where the performance of gender/sex is unobserved. Even in the absence of others, the self—with its ingrained binary views of gender/sex and internalized gender norms—is always watching and informs the performance of gender/sex (for literature on self-stereotyping, see, e.g., Coleman & Hong, 2008; Latrofa, Vaes, Cadinu, & Carnaghi, 2010).

When gender/sex is not performed “correctly,” the audience can react in a variety of ways, from feeling threatened and reacting defensively to embracing the gender trouble or changing their views of gender. These reactions depend on a range of factors, including (a) individual psychological factors such as political ideology, beliefs in gender/sex essentialism, or the need for cognition (Norton & Herek, 2013; Stern & Rule, 2018; Tebbe & Moradi, 2012; Wilton et al., 2019); (b) group-based factors such as in-group status and group identification (C. T. Nagoshi et al., 2019); and (c) contextual factors such as gender/sex salience and norms (Levitt, 2019). But what exactly do we mean by gender trouble?

**Fig. 1.**
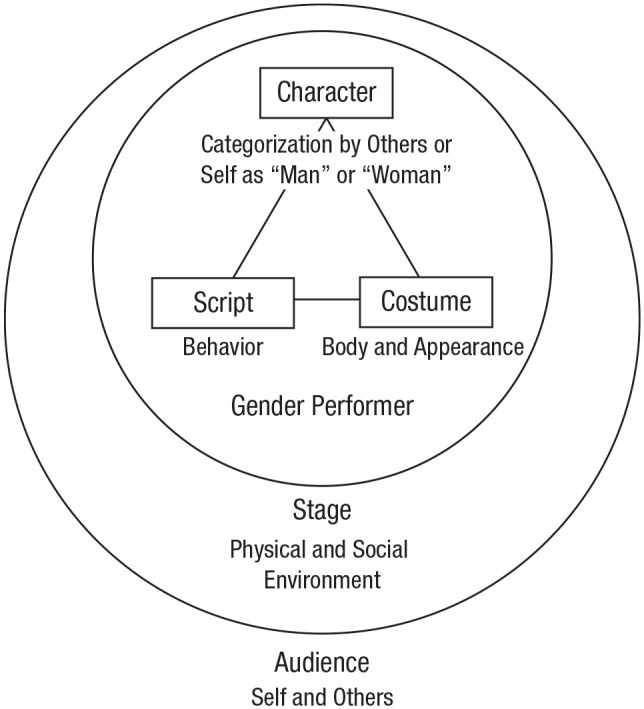
Elements of binary gender/sex performance.

We propose that the construction of gender/sex as binary and essential necessitates the stable *alignment* of character, costume, and script and that the stage is set up to facilitate this alignment (see Fig. 1). Alignment occurs when those who identify (or are identified by others) as women look feminine (including having the “right” set of genitals) and act in feminine ways. This includes being sexually attracted to and engaging in sexual acts with men, but also the display of nurturing and warm behaviors and feminine interests. Likewise, those who identify (or are identified) as men are expected to look masculine and act in masculine ways (see Deaux & Lewis, 1984).

Disruption (i.e., gender trouble) occurs when one of these core elements does not align with the other two, for example, a woman acting assertively or a man looking feminine. When looking at Figure 1, it might be useful to imagine gender/sex as a three-legged stool: If one of the legs does not fit with the others, the whole construct of gender/sex can become unstable.

We propose that when disruption occurs, adjustments are made—if possible—to reconcile the three different elements (see Deaux & Lewis, 1984). Going back to the three-legged stool analogy, to fix this precarious situation and reestablish stability, measures have to be taken to either realign the leg that is causing trouble or to lengthen or shorten the other legs accordingly. To give an illustrative historical example, in Western countries, trousers were considered clearly male clothing until the early 21st century, and laws prohibited women from wearing them in many places (Drover, 2017). Trousers were thus clearly part of a man’s, but not a woman’s, costume, and women who wore them did indeed cause gender trouble. However, as more and more women started wearing trousers in the 1920s, either for practical or political reasons, this perception slowly changed, and today they are, for the most part, seen as a gender-neutral item (Steinmetz, 2016). Thus, the imbalance created by women wearing a “male” costume was addressed by changing the way that trousers were viewed. In addition, the gender/sex binary is reinforced by ensuring that women’s and men’s trousers can be distinguished through different cuts, the inclusion of pockets or pocket sizes, and the direction of buttons.

To give a more current example, we argue that transgender women and men are likely to evoke pressure from the audience to also change their costume and their scripts in line with the perceived change in character (see J. L. Nagoshi, Brzuzy, & Terrell, 2012). For example, if someone who was assigned the character of a man by society identified as a woman (as would be the case for trans women) and thus switched the character (at least in the eyes of those who categorized her as male), corresponding changes in the costume and script would also be expected by the audience (see J. L. Nagoshi et al., 2012). If the trans woman in question refused to act in stereotypically feminine ways (including attraction to men, not women) did not alter her body (e.g., by undergoing gender confirmation surgery and/or hormone replacement therapy, removing body and facial hair) and her appearance more generally (e.g., by wearing makeup and women’s clothing), her character would not align with her costume and script in the eyes of the audience. This misalignment, this gender trouble, poses a threat to the gender/sex binary that would need to be resolved, for example, by denying her identity and continuing to categorize her as male (Friedman, 2014). If she wanted her identity as a woman to be acknowledged, she would be expected to perform her gender/sex “correctly” by putting on the “correct” costume and following the “correct” script.

Another strategy to realign character, script, and costume is to pressure the gender/sex performer into realignment. Open hostility, discrimination, ostracism, and violence are all strategies that are frequently used in this way. For example, masculine-appearing lesbians (i.e., those whose costume does not match their character and script) experience higher levels of discrimination, threats of violence, and actual violence than feminine-appearing lesbians; Levitt, Puckett, Ippolito, & Horne, 2012). Likewise, although trans individuals in general face high levels of discrimination, the discrimination that gender-nonconforming trans individuals face is even more pronounced (Miller & Grollman, 2015). Last, even women and men for whom character and costume align face backlash when they deviate from their assigned script (Moss-Racusin et al., 2010; Rudman, Moss-Racusin, Phelan, & Nauts, 2012).

These acts of alignment that maintain the gender/sex binary take place both on the side of the one performing gender, altering scripts and costume to fit with one’s character (sometimes as an authentic expression of one’s gender identity, sometimes as a necessary tool for conveying one’s gender identity to others; see J. L. Nagoshi et al., 2012), and on the part of the audience, biasing perception and shaping reactions to the performer of gender/sex (see Deaux & Major, 1987; Eagly & Wood, 2012). However, the extent to which this alignment takes place depends on the nature of the audience. For example, LGBTQ communities have generally been more open to misalignment (see Levitt, 2019), and thus performances in front of LGBTQ audiences are often less restricted by these binary norms (Mann, 2011). That said, even in these communities, heteronormative, essentialist, binary views of gender/sex have sometimes been reinforced, albeit to a lesser extent than in society more generally. For example, in lesbian communities that developed in the United States in the second half of the 20th century, women were generally expected to identify and perform the role of either *butch* (i.e., a masculine lesbian) or *femme* (i.e., a feminine lesbian) and to date women who performed the “opposite” role (see Levitt, 2019). In parts of the lesbian community, similar patterns can still be observed today (Panesis, Levitt, & Bridges, 2014; Rothblum, Balsam, & Wickham, 2018). Likewise, Napier, van der Toorn, and Vial (2019) found that gay men often seek “complementary” partners in line with heteronormative ideals (i.e., gay men who perceive themselves as more feminine in terms of gender roles show a preference for more masculine men and vice versa). Interestingly, and in line with the predictions of our model, this was particularly the case among gay men with high levels of internalized stigma for whom discrimination was salient. In other words, these gay men voiced partner preferences that can be seen as a partial realignment between the elements described above—in which the feminine script includes both feminine behaviors and attraction to masculinity, whereas the opposite is the case for the masculine script.

The stage is also set in a way that helps the realignment of character, costume, and script. It consists of many different elements that can reinforce the gender/sex binary, including physical spaces (e.g., gender/sex-segregated bathrooms and classrooms, stores and brands that organize and label their products in gendered ways), language (e.g., gendered pronouns, grammatical gender), the media (e.g., portrayal of women and men, representation of trans and nonbinary people), and laws (e.g., how many genders/sexes are legally recognized, the presence of gender/sex on legal documents, and gendered legislation, such as that regarding parental leave or military service). For example, research has found that laws and cultural norms are associated with identity formation and decisions of those from gender minorities (Katz-Wise et al., 2017). In other words, identifying as transgender or nonbinary is much easier when these are accepted identities and when there is a policy framework that recognizes them legally.

It is important to note that the stage is not set this way by chance. Binary, essentialist views underlie the construction of the stage, as it serves to buttress the gender/sex binary. In line with these views, Roberts, Ho, Rhodes, and Gelman (2017) showed that people who held highly essentialist beliefs about gender/sex were more supportive of policies and practices that reinforce the gender/sex binary such as gender/sex-segregated classrooms and legislation forcing trans people to use the bathroom associated with the sex they were assigned at birth. As such, it can be argued that such policies are specifically put in place to fit with essentialist views of gender/sex (see also Wilton et al., 2019). Thus, like the binary performance of gender, the stage has an important function in reinforcing the gender/sex binary.

**Fig. 2.**
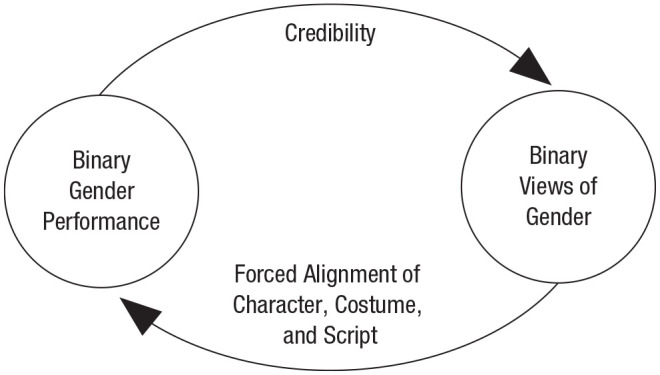
The self-reinforcing cycle of the gender/sex binary.

We argue that such effortful alignment would not be necessary if the construction of gender/sex were not so narrowly defined or if it were policed less heavily. The binary performance of gender/sex creates a self-reinforcing cycle (see Fig. 2). Here the presence of binary categories necessitates distinct and visible differences between them to justify, and give credibility to, their very presence. The binary, essentialist construction of gender/sex leads to the enhancement of gender/sex differences in which characters wear the correct costumes and follow the correct scripts (see Eagly & Wood, 2012; Hill & Willoughby, 2005; Roberts et al., 2017) and is in turn reinforced by the resulting performance of gender, which acts as a “proof” that there are two genders/sexes that differ in important ways.

These processes mirror Butler’s ideas of gender performativity. Butler argues that gender/sex is created by its own performance—through repeated, gendered, socially sanctioned acts—and hence it is performative. The term “performativity” was originally used by Austin (1962) in relation to utterances that create the very thing they describe. For example, the sentence “I declare war” not only describes what the person is doing (i.e., declaring something) but also creates the war the person is declaring through the act of the declaration. Butler applies the same principle to gender, arguing that gender is created by its own repeated performance. However, the ubiquity of the binary performance of gender/sex masks its performative nature and makes it appear natural. These processes are further reinforced by social sanctions faced by those who disrupt the gender/sex binary.

Although performativity is not generally used as an actual term in psychology, the idea is very much in line with established psychological theory. For example, social-role theory (Eagly, 1987; Eagly & Wood, 2012) as well as the stereotype-content model (Fiske, Cuddy, Glick, & Xu, 2002) proposes that societal structures (e.g., power, division of labor) are at the root of gender stereotypes that affect both gendered behavior and appearance (i.e., script and costume) and the reactions to those who perform gender/sex—which in turn reinforces the societal structures and binary views of gender/sex (see Morgenroth & Ryan, 2018).

However, although the self-reinforcing cycle fortifies the gender/sex binary, it also demonstrates the potential for disruption and subversion through what Butler (1990) calls “repeated reconfiguration.” If gender/sex is repeatedly performed in ways that make the alignment of character, costume, and script impossible—or at least more difficult—the gender/sex binary should, over time, become less and less convincing and lose its regulatory power. Such gender trouble could thus result in a change to the available characters themselves (e.g., having more than two genders/sexes, having a less fixed or essentialist understanding of gender/sex), a change in scripts and costumes (e.g., less pronounced gender stereotypes, more flexible gender roles), and changes to the stage (e.g., less emphasis on gender/sex in society, a context that allows more than two genders/sexes).

In summary, we outlined a framework explaining the perpetuation of the gender/sex binary and the potential for disruption, building on Butler’s work on gender performativity as well as Goffman’s work on gender as a performance. This framework identifies important aspects of the performance of gender/sex and how they work together to perpetuate the gender/sex binary. We argued that (a) the alignment of character, costume, and script reinforces the gender/sex binary, and, at the same time, and (b) binary views of gender/sex lead to the alignment of character, costume, and script. We further proposed that the stage plays an important part in the performance of gender/sex, as it can highlight or obfuscate binary and nonbinary performances of gender. Last, we argued that gender trouble could lead to an erosion of the gender/sex binary. However, despite the potential for gender trouble, such disruption is likely to lead to threat and be met with resistance and efforts to reinforce the gender/sex binary, as we outline below. First, however, we provide more detail and further examples of different types of gender trouble.

### The different forms of gender trouble

We broadly distinguish between two categories of gender trouble: performance-based gender trouble, which can be category-based (playing a different character), appearance-based (putting on a different costume), or behavior-based (deviating from the script); and context-based gender trouble (dismantling the stage). It should be noted that some forms of gender trouble do not fall clearly within one of these two categories. However, most examples are primarily category-based, appearance-based, or behavior-based, but, as these facets are closely linked, the lines between them are often blurry. Nevertheless, it is useful to distinguish between them because, in some cases, reactions to them can differ. For example, research indicates that reactions to androgynous behavior are quite different from reactions to androgynous appearance (see Stern & Rule, 2018).

#### Performance-based gender trouble

A diverse range of identities, behaviors, and appearances can cause gender trouble, including deep-seated and stable identities (e.g., trans and nonbinary identities), gender nonconforming and counterstereotypical behavior (e.g., women acting assertively or taking on leadership positions, men acting modestly or staying home with their children, expressions of same-sex desire), and androgynous or gender-nonconforming appearance (e.g., crossdressing and drag; men wearing feminine jewelry, skirts, or makeup; women having short hair or wearing ties). Note that each of these forms of gender trouble can lead to negative reactions from the audience that vary in their severity (from mild forms of teasing to extreme violence), meaning that not everyone is free to cause gender trouble without risk. In addition, gender performers have little control over some forms of gender trouble (e.g., their gender identity or to whom they are attracted, personality traits), whereas others can be more freely chosen (e.g., how to dress or how to express one’s personality). Thus, although we agree with Butler’s call to cause gender trouble, we also acknowledge that there are limits on the extent to which this is possible that depend on context and the type of gender trouble.

Each type of performance-based gender trouble can broadly take two forms. The first is switching to the “opposite” character (e.g., trans people), costume (e.g., drag performers), or behavior (e.g., sexual attraction in gay and lesbian people). The second is performing a character, putting on a costume, or following a script that is neither clearly male/masculine nor female/feminine but instead a mix of both or outside of these categories altogether (e.g., nonbinary identities, androgynous appearance, bisexuality). These two forms are both disruptive, albeit for different reasons. Switching to the opposite character, costume, or behavior leads to misalignment between the three elements as described above. Performances that do not follow binary structures at all, on the other hand, make it more difficult to force individuals into binary categories. For example, should an androgynous-appearing individual be categorized as women or men?

Switching to the opposite character, costume, or script can be disruptive, even when the three elements are aligned. For example, trans men who present and act in very masculine ways nevertheless are clear evidence that gender does not always follow from sex (as the gender/sex binary claims), and drag queens who look overly feminine and adhere to feminine gender stereotypes during their performance question the stability of gender given that they are generally perceived and responded to as women when in drag but as men when out of drag. In both of these cases, the innateness and immutability of gender/sex alleged by the gender/sex binary is challenged.

Any form of performance-based gender trouble can also be either permanent (e.g., altering one’s body permanently through gender confirmation surgery) or temporary (e.g., crossdressing for one night), both of which can be disruptive in different ways. On the one hand, permanent gender trouble is likely to evoke strong reactions. When gender trouble is temporary, cognitive heuristics such as the confirmation bias (Wason, 1960) may help the audience to overlook disruptions, keeping character, costume, and script aligned with one another. For example, individuals are more likely to remember stereotype-congruent information and distort information that contradicts gender stereotypes in their memories (Fyock & Stangor, 1994). Thus, these heuristics reinforce preexisting beliefs (e.g., that a person who is categorized as male looks and acts masculine). On the other hand, temporary gender trouble is likely to challenge essentialist views of gender/sex (i.e., that character, costume, and script are aligned in an immutable way) and in this way may be disruptive to the gender/sex binary.

The way in which individuals engage in performance-based gender trouble depends on multiple factors. One of the likely main determinants is the degree to which these performances feel authentic. Individuals generally have a strong and stable sense of gender identity and use costume and script to express this identity (see Levitt, 2019). Likewise, identifying with other, intersecting identities can alter gender performance (i.e., the costume and script may look different for women and men of different racial and ethnic groups). At the same time, the audience as well as the stage can encourage or discourage performance-based gender trouble. For example, research indicates that drag queens adhere more strongly to binary gender/sex norms when performing for a heterosexual audience (e.g., by portraying exaggerated femininity) than an LGBTQ audience, where they are more likely to mix women’s and men’s costumes (e.g., by wearing a dress but displaying body hair; see Mann, 2011).

To summarize, performance-based gender trouble can take a range of forms: switching character, costume, or script in a way that leads to misalignment; playing a character, putting on a costume, or enacting a script that is neither clearly male/masculine nor clearly female/feminine; or switching characters, costumes, and scripts in a way that is still aligned but questions the immutability and innateness of gender/sex.

#### Context-based gender trouble

As we mentioned above, the performance of binary gender/sex takes place on a stage that is set up to highlight its binary nature and obfuscates gender trouble (see Roberts et al., 2017). We argue that there are two potential strategies to dismantling the stage—*degendering* the context and *multigendering* the context, similar to the distinction Bem (1995) makes between “turning the volume down or up.” Degendering refers to strategies that aim to decrease the salience and importance of gender/sex (turning the volume down) by removing gender/sex division and gender/sex cues from different contexts. For example, language can be degendered by using the pronoun “they” to refer to all genders/sexes, or space can be degendered by providing individual bathroom stalls that are ungendered, and legislatively it might be the removal of gender from passports. To the extent that these strategies indeed decrease gender/sex salience, they may, however, leave the binary system of gender/sex unchallenged. In other words, not thinking about gender/sex, by definition, implies not questioning ideas about gender/sex. For example, research suggests that gender-neutral (i.e., degendered) language such as “they” or “the candidate” are often just processed as male, in line with androcentric “male-as-default” assumptions (Lindqvist, Renström, & Gustafsson Sendén, 2019).

Multigendering, on the other hand, refers to strategies that aim to disrupt the gender/sex binary by bringing attention to genders/sexes outside of the binary (turning the volume up). For example, for language this might include the introduction of new pronouns such as “ze” for nonbinary people, for space this might be the addition of an all-gender bathroom, and legislatively this might include allowing individuals to select a third gender on their passport. Thus, although such multigendering strategies are likely to make gender/sex salient such strategies, they will at the same time highlight its nonbinary nature.

### The psychology of the audience’s reaction to gender trouble

Although it may seem as if the behaviors of gender trouble we described above have little in common, we argue that they are similar in that they all have the potential to threaten the same system—the gender/sex binary—and, therefore, reactions to these behaviors are likely to take similar forms. In this section we explore potential reactions to different forms of gender trouble and some of the psychological mechanisms that may contribute to the perpetuation of the gender/sex binary. More specifically, we argue that gender trouble can elicit different forms of threat in audience members (C. T. Nagoshi et al., 2019; Outten, Lee, & Lawrence, 2019), which, in turn, may prompt the reinforcement of the gender/sex binary through various psychological processes. These processes include (a) cognitive efforts to realign character, costume, and script, including the stereotypical subtyping of troublemakers and motivated cognition such as biased information processing and memory (e.g., E. L. Haines & Jost, 2000); (b) increasing the endorsement of system-justifying beliefs such as benevolent sexism or gender essentialism (e.g., Brescoll, Uhlmann, & Newman, 2013); (c) gender stereotyping and conformity to gender stereotypes (e.g., Laurin, Kay, & Shepherd, 2011); (d) negative attitudes toward, and dehumanization of, gender troublemakers (e.g., Garelick et al., 2017); (e) discrimination and punishment of gender troublemakers, ranging from social and economic penalties to violence, including murder (e.g., Human Rights Campaign, 2018; Rudman, Moss-Racusin, Glick, & Phelan 2012); (f) delegitimization of gender troublemakers and denial of their identity (e.g., Brewster & Moradi, 2010; Friedman, 2014); and (g) the endorsement of policies that strengthen the gender/sex binary and opposition to attempts to dismantle the stage (e.g., Outten et al., 2019; Roberts et al., 2017; Zingora & Graf, 2019).

Drawing primarily on a social-identity approach (Tajfel & Turner, 1979; Turner, Hogg, Oakes, Reicher, & Wetherell, 1987), intergroup threat theory (Stephan, Ybarra, & Morrison, 2009), and system justification theory (Jost & Banaji, 1994), we argue that the gender/sex binary helps fulfill a range of psychological needs. It gives individuals important identities and group memberships, providing them with a sense of belonging, a source of self-esteem, and a sense of who they are (and who they are not). As gender/sex is one of the most important social identities (Deaux, 1991), individuals are particularly motivated to protect their gender/sex group and the concept of gender/sex as a categorizing variable. The gender/sex binary also establishes a hierarchical system that provides power and status to some while withholding it from others (see Butler, 1990). Moreover, it provides certainty, predictability, and stability by creating and protecting a system of oppositional, distinct gender/sex identities (see Brescoll et al., 2013), as well as the relationship between gender/sex groups in a seemingly complementary and mutually beneficial fashion (Glick & Fiske, 1996). Thus, it can provide benefits for the self, for one’s group, and for the functioning of society as whole.

As we describe in detail below, gender trouble threatens this system and the benefits it purportedly provides to individuals, to their in-groups, and to society as a whole. As individuals are motivated to protect and advance the interests of the *self*, their *group*, and the *system* or culture they are part of (e.g., Jost & Banaji, 1994; Tajfel & Turner, 1979), any challenge to the gender/sex binary can evoke threat. We argue below that different forms of gender trouble can evoke *personal threats, group and identity threat*s, and *system threats* and, in turn, efforts to alleviate these threats in the audience. The type and level of threat are dependent on the type of gender trouble as well as a range of contextual and individual factors, which are summarized in Table 1.

**Table 1.** Threat Reactions to Gender Trouble

Type of threat	Type of gender trouble—particularly likely evoked by . . .	Audience—particularly pronounced for . . .	Reactions
Personal threats			
Personal status threat	Some forms of character-based gender trouble Some forms of script-based gender trouble Degendering the stage	Men who identify strongly with their gender/sex Men who hold essentialist views of gender/sex Men who define masculinity in traditional terms	Gender stereotyping and conformity to gender stereotypes Negative attitudes toward gender troublemakers Discrimination and punishment of gender troublemakers Delegitimization of gender troublemakers Endorsement of policies that strengthen the gender/sex binary and opposition to attempts to dismantle the stage
Safety threat	Degendering the stage Some forms of character-based gender trouble	Women Benevolent sexist men (on behalf of women) Those with essentialist views of gender/sex	Negative attitudes toward gender troublemakers Discrimination and punishment of gender troublemakers Endorsement of policies that strengthen the gender/sex binary and opposition to attempts to dismantle the stage
Group-based and identity threats			
Distinctiveness threat	Gender troublemakers “outside of the binary” Degendering and multigendering the stage	Women and men who are highly identified with their gender/sex Marginalized groups (e.g., women, lesbians)	Increase in the endorsement of system-justifying beliefs Gender stereotyping and conformity to gender stereotypes Negative attitudes toward gender troublemakers Discrimination and punishment of gender troublemakers Delegitimization of gender troublemakers and denial of their identity Endorsement of policies that strengthen the gender/sex binary and opposition to attempts to dismantle the stage
Group-based-status threat	Some forms of character-based gender trouble Some forms of script-based gender trouble Some forms of costume-based gender trouble	Men who highly identify with their gender/sex Men who highly identify with right-wing authoritarianism Men high in social-dominance orientation Gay men with traditional views of masculinity	Gender stereotyping and conformity to gender stereotypes Negative attitudes toward gender troublemakers Discrimination and punishment of gender troublemakers Delegitimization of gender troublemakers Endorsement of policies that strengthen the gender/sex binary and opposition to attempts to dismantle the stage
System threat	Any kind of gender trouble	Individuals who feel dependent on system Individuals low in need for cognition Individuals high in death anxiety Individuals with high need of shared reality Conservatives Feminists with essentialist views of gender/sex	Cognitive efforts to realign character, costume, and script Increase in the endorsement of system-justifying beliefs Gender stereotyping and conformity to gender stereotype Discrimination and punishment of gender troublemakers Endorsement of policies that strengthen the gender/sex binary and opposition to attempts to dismantle the stage

Note: This table gives an overview of the types of gender trouble that are likely to elicit threat and the potential reactions. For concrete examples, please see the text. The type of gender trouble, audience, and reactions listed in the same row do not necessarily indicate that they are strongly linked; the same reaction or audience can be linked to multiple forms of gender trouble.

#### Personal threats

We argue that there are two main types of personal threat that gender trouble can evoke in members of an audience. For men, it is likely to threaten their manhood and in turn their individual status and power, whereas for women it may threaten perceived bodily safety. Whereas the former can be categorized as a form of *symbolic* individual threat, the latter constitutes a *realistic* individual threat according to intergroup-threat theory (Stephan et al., 2009). Symbolic individual threats are concerned with a loss of face, honor, or self-esteem, whereas realistic individual threats are more about physical or material harm. Although both types of threat are likely to result in negative attitudes and behaviors toward the troublemakers, reactions may nevertheless differ. Stephan and colleagues (2009) argue that symbolic threats are more likely to lead not only to particularly strong behavioral responses such as violence but also to dehumanization, delegitimization, and reduced empathy. Symbolic threats are also more likely to lead to in increased conformity to the group norms. Realistic threats, on the other hand, may lead to primarily behavioral responses aimed at reducing the threat such as withdrawal, negotiation, but also aggression, depending on the status of the out-group.

##### Personal-status threat

Gender trouble has the potential to threaten an audience member’s status, particularly the status of men in the eyes of other men, by threatening their manhood (Vandello, Bosson, Cohen, Burnaford, & Weaver, 2008). The idea that manhood is something that has to be achieved and can be lost—and must therefore be proven repeatedly (in line with Butler’s conception of gender performativity)—has been noted repeatedly by scholars and has been demonstrated in the literature on *precarious manhood* (for a review, see Bosson, Vandello, & Caswell, 2013). The authors (e.g., Vandello et al., 2008) demonstrate that in order to be a “real man,” men must continually prove their manhood, especially in front of other men, by actively performing masculinity and avoiding anything deemed feminine. Gender trouble can threaten this performance in a range of ways. For example, script-based gender trouble such as women in leadership positions can threaten men’s status by occupying masculine positions in society (Netchaeva, Kouchaki, & Sheppard, 2015). Likewise, attempts to dismantle the stage may make it more difficult to discern what is masculine and what is feminine, making it harder to perform masculinity.

We argue, however, that appearance-based and character-based gender trouble are particularly threatening. Because heterosexuality is a core part of masculinity (Herek, 1986) and gay men are perceived to be more similar to women than to straight men (Kite & Deaux, 1987), experiencing same-sex desire—or being perceived as gay—is highly threatening (Kroeper, Sanchez, & Himmelstein, 2014). Trans women therefore pose a particular potential threat to the masculinity of heterosexual men who hold essentialist, fixed views of sex and gender and thus view trans women as men “dressing up as” women. We propose that this view can lead to heterosexual men perceiving that trans women are “tricking” them into (in their view) same-sex desire and behavior, which threatens their manhood. Take, for example, the case of Gwen Araujo, a transgender teenage girl who was murdered by four men in 2002 after flirting with them and engaging in sexual acts with two of them. After discovering she was transgender, one of her killers cried “I can’t be fucking gay” before beating her to death (Lee, 2003). This illustrates how the desire to appear heterosexual—particularly in front of other men—can have devastating consequences for those who dare challenge the gender/sex binary. Of note in this case—and other similar cases—is the role of ethnicity and race. More specifically, extreme acts of violence disproportionately affect trans women of color (particularly Black and Latina women), in whose communities masculinity norms are often particularly strongly endorsed (see Levant & Richmond, 2007), likely as a response to their marginalization (see Majors & Billson, 1992).

Gwen Araujo’s case is also an illustration of the findings that men whose manhood has been threatened are more likely to engage in behaviors that are seen to reinforce their masculinity, such as violence (see Bosson et al., 2013). Likewise, it is in line with the prediction by intergroup-threat theory that symbolic threat should lead to a stronger adherence to in-group norms (Stephan et al., 2009). These processes may take place even in the absence of physical attraction, as men may be concerned that any affiliation with trans women will be judged by other men as same-sex desire, similar to affiliation with gay men (Herek, 1986).

For heterosexual women, for whom proscriptions regarding same-sex desires and acts are less strict (C. T. Nagoshi et al., 2019) and who do not have to prove their “womanhood” in the same way as men (Vandello et al., 2008), the thought of having a same-sex sexual encounter should be less threatening. In line with this expectation, C. T. Nagoshi and colleagues (2019) demonstrated that transphobia is indeed particularly high for cis men when judging trans women. Among women, transphobia is generally lower and does not differ depending on the gender/sex of the troublemaker (Makwana, Dhont, Akhlaghi-Ghaffarokh, Masure, & Roets, 2018; C. T. Nagoshi et al., 2019).

Threat experienced by men is likely more pronounced among certain men. First, men who identify strongly with their gender/sex are likely to care more about other men’s views of them. Moreover, men who hold traditional, binary, essentialist views of gender/sex view masculinity in traditional terms and might in turn feel that trans women threaten their manhood to a higher extent. In line with this view, research indicates that such men exhibit higher levels of prejudice against trans people (Norton & Herek, 2013; Tebbe & Moradi, 2012).

##### Safety threat

Challenges to the gender/sex binary may also induce safety threats, a type of realistic threat according to intergroup-threat theory (see Stephan et al., 2009), particularly the (perceived) safety of women and children. The concern for women’s safety might be prevalent among both men and women, albeit for different reasons.

We argue that women may experience safety threat, as they are more often targets of intergroup violence such as sexual violence or domestic violence (Smith et al., 2018), which clearly form a threat to their safety from the out-group (i.e., men). In turn, women have legitimate concerns about their safety and want to protect women-only spaces in which they can be safer from male violence. These spaces include bathrooms, changing rooms, prisons, and women’s shelters. Blurring of the boundaries between men and women can be interpreted as a threat when women believe that these changes will enable male aggressors to enter women-only spaces (e.g., men assaulting women in unisex bathrooms; e.g., Stock, 2018). In turn, some may engage in efforts to reinforce the gender/sex binary. These responses are likely to be the most pronounced in reaction to attempts to dismantle the stage (i.e., context-based gender trouble) and more specifically to degendering rather than multigendering spaces (see Outten et al., 2019).

However, to the extent that members of an audience believe that gender is an essential quality stemming directly from sex assigned at birth, category-based gender trouble (i.e., trans people and nonbinary individuals) may elicit similar reactions, such as concerns that trans women—who are seen by these individuals as men—will enter women-only spaces and pose a threat to them. Although the concern that trans women pose a threat to women’s safety is not uncommon among cis women (Trotta, 2016), research indicates that it is more pronounced among cis men, who in turn voice more concern for women’s safety (Stones, 2017). We argue that when men voice this concern, it is less likely to stem from legitimate safety concerns for women and more likely to be an expression of either (a) benevolent sexism (see Blumell, Huemmer, & Sternadori, 2019) or (b) threat to their own status (as described above and below) as well as the system of society itself, disguised as an altruistic concern for women in order to seem more legitimate.

The idea that women need to be protected by men—in this case from either cis men or transgender women who are perceived as men entering women-only spaces—is one of the core beliefs of benevolent sexism (Glick & Fiske, 1996). Benevolent sexism, as the name implies, is accepted much more widely in society and endorsed more strongly than other forms of sexism, even by women (Barreto & Ellemers, 2005; Glick & Fiske, 1996). Voicing concerns for women’s safety may therefore seem like a legitimate and honorable concern when voiced by men. However, rather than being protective of women who experience sexual violence, benevolent sexism has been linked to higher assignments of blame to rape victims who violate gender role expectations (Viki & Abrams, 2002). In other words, we argue that men’s concerns about women’s safety may be a tool that is used to disguise threats to their own status and to keep the current gender/sex system intact.

We propose that similar processes are at play when it comes to children’s safety (see Herek, 2002a). Here, the concern more often seems to be about their emotional, psychological, or moral safety rather than their physical safety. This process is illustrated by reactions to programs such as Drag Queen Story Hour, in which drag queens read stories to children at libraries. Although these programs have proven very popular and successful, they have also faced backlash from conservatives with calls to “protect the children” (Sharp, 2018). Similar arguments are often voiced in response to the inclusion of LGBTQ content in schools (Parveen, 2019). Here again, we argue that it is not likely to be true concerns about children’s physical safety that drives these reactions but threat to one’s own values and the current system of gender/sex—as we describe in the section on system threats below.

In summary, we have argued that there are cases in which gender trouble can lead to safety concerns for women, particularly when women-only spaces are threatened. However, in the majority of cases in which concerns about safety are voiced, these may be merely a convenient disguise for other, less altruistic, types of threats.

#### Group and identity threats

In addition to threatening individual status and safety, disruptions to the gender/sex binary also have the potential to induce group-based threats in the audience, either in terms of group-based identities or in terms of the resources and power available to gender/sex groups. More specifically, gender trouble can elicit *distinctiveness threat*, that is, threat to the clear differentiation between women and men (Branscombe, Ellemers, Spears, & Doosje, 1999) and the benefits these social identities provide (Outten et al., 2019). Moreover, it can threaten the status of men as a group (C. T. Nagoshi et al., 2019).

##### Distinctiveness threat

A central tenet of the social-identity approach (Tajfel & Turner, 1979; Turner et al., 1987) is that members of groups—including men and women—have a need to see their own group as distinct and different from the out-group. The gender/sex binary and the alignment of character, costume, and script serve this need well, as they enhance the contrast between the two groups. A stage that enhances the visibility of two opposing genders/sexes similarly serves this need. Gender trouble, on the other hand, can potentially blur the boundaries between women and men (i.e., make the group boundaries more permeable) and thus threaten the clear distinction between—and legitimacy of—these two categories (see Outten et al., 2019). In turn, such gender trouble is likely to provoke a range of negative reactions among women and men, particularly among those who are highly identified with their gender/sex (e.g., Schmitt & Branscombe, 2001). It is important to note that these reactions might differ depending on whether the gender troublemaker is an in-group or an out-group member. The literature on the “black sheep effect” suggests that reactions might be particularly negative when a perceived in-group member is not adhering to group norms (Marques, Yzerbyt, & Leyens, 1988). Thus, men may react more strongly to male gender troublemakers (or whom they perceive as male), that is, men who dress or act in feminine ways, but also gay or bisexual men or trans women, whereas women may react more strongly to female gender troublemakers (or whom they perceive as female), that is, women who dress or act in masculine ways, but also lesbian and bisexual women as well as trans men. At the same time, the literature on impostors (Hornsey & Jetten, 2003) demonstrates that perceived out-group members who “pretend” to be in-group members are also threatening. In the context of gender, this means that cis women with binary, fixed views of gender/sex may react particularly negatively to trans women who they see as illegitimately trying to join their group, whereas the opposite may be the case for cis men.

We argue that both cases—perceived deviant in-group members and impostors—threaten the distinctiveness of gender/sex groups, and this is also the case for women and men with intersecting identities who do adhere to gender norms but who may perform their gender differently (e.g., people of color, members of sexual-minority groups). The same is true for attempts to degender the stage, for example, unisex bathrooms or gender-neutral language, or to multigender the stage, for example, by offering a third gender option on official documents, as a third group makes a clear distinction between two oppositional identities harder. Reactions to distinctiveness threat can include identity uncertainty (feeling uncertain about what it means to be a man or woman), which has been shown to be associated with lower collective self-esteem and higher collective angst and anger (Wagoner, Belavadi, & Jung, 2017). We argue that highly identified women and men may therefore be motivated to reduce distinctiveness threat by reinforcing the gender/sex binary in multiple ways that include their own gender/sex performance (Branscombe et al., 1999), their views of gender/sex such as increased essentialism (Falomir-Pichastor & Hegarty, 2014), their reactions to gender troublemakers (Branscombe et al., 1999), and their attempts to dismantle the stage (Outten et al., 2019).

With regard to their own gender/sex performance, individuals may increase intergroup contrast by endorsing and adhering to gender/sex stereotypes in a way that maximizes gender/sex differences. For example, individuals may put on the costume and follow the script associated with their own gender/sex and avoid those costumes and scripts of the opposite gender/sex (Branscombe et al., 1999). We further propose that distinctiveness threat may lead to a stronger endorsement of essentialism. Findings on strategic essentialism suggest that essentialism is not a stable construct but serves a range of identity-related functions can be strategically endorsed or rejected to fulfill these functions (Falomir-Pichastor & Hegarty, 2014; Hoyt, Morgenroth, & Burnette, 2019; Morton & Postmes, 2009), such as when an important identity is marginalized. This might be particularly pronounced for women, as they, compared with men, more often experience marginalization in society. Although we know of no psychological evidence for this argument, it is illustrated by recent voices from a subgroup of feminists claiming that trans women threaten the notions of womanhood (Williams, 2016) or of gay women, who arguably face even more marginalization, claiming that trans women are “erasing” lesbians (Earles, 2019).

Although the increase in essentialism in response to distinctiveness threat is an interesting outcome in itself, it could also affect reactions to gender troublemakers, particularly when gender trouble is character-based. More specifically, distinctiveness threat may lead to the denial of identity to nonbinary and trans people by claiming binary gender/sex categories are inherent, essential, biological, and fixed (i.e., by essentializing gender/sex), rendering any identity in between these categories as either impossible, fleeting, or abnormal (e.g., as a mental disorder; see Howansky, Wilton, Young, Abrams, & Clapham, 2021) and thus decreasing their potential to disrupt the gender/sex binary.

This essentialization can arise in response to trans identities, particularly when script and costume do not align with the character, but we propose that it is even more likely for nonbinary identities (see McLemore, 2015). Likewise, bisexual individuals are likely targets for identity denial. The gender/sex binary also conceptualizes sexuality as a dichotomy, with gay/lesbian and heterosexual as the only available identities (Eliason, 1997). Bisexual individuals disrupt this dichotomy of sexuality and threaten not only the clear distinction of what it means to be a man (i.e., being attracted exclusively to women) and to be a woman (i.e., being attracted exclusively to men) but also the distinctiveness of binary sexual identities (i.e., heterosexual vs. gay/lesbian). Denying their existence (e.g., by claiming that a bisexual woman is just kissing other women for attention from heterosexual men or that a bisexual man just has not yet come out as gay; see Brewster & Moradi, 2010) alleviates this threat and reinforces the binary. Bisexual individuals face this identity denial not only from heterosexual individuals but also from the LGBTQ community itself (Burke & LaFrance, 2018; Mohr & Rochlen, 1999). Likewise, attitudes toward bisexuals, particularly toward bisexual men, are more negative than those toward lesbian and gay individuals (Helms & Waters, 2016; Herek, 2002b) among heterosexual as well as gay and lesbian individuals (Mulick & Wright, 2002). These reactions can be seen as a way of eliminating the threat bisexuality poses to the gender/sex binary.

Finally, we propose that distinctiveness threat may result in opposition to attempts to dismantle the stage. This opposition is likely to be particularly pronounced in reaction to attempts to degender the context, as these strategies directly aim to abolish gender/sex categorization. For example, in the context of sexual orientation, Schmitt, Lehmiller, and Walsh (2007) found that heterosexual Americans were much more opposed to same-sex marriage policies compared with civil-union policies, even if the content of the policies was otherwise the same, and this was because of perceptions that the same-sex marriage policy was more threatening. This example illustrates how policies can be used to reinforce group boundaries and that attempts to blur these boundaries are met with resistance. The participants in Schmitt and colleagues’ studies also reported that boundary-blurring policies threatened their status, an issue we turn to next.

##### Group-based-status threat

We have argued that gender trouble can threaten individual men’s masculinity and, in turn, their personal status. Here we argue that it can also threaten men’s status as a group by undermining the patriarchy and can therefore lead to similar reactions (see Butler, 1990; C. T. Nagoshi et al., 2019). In line with Butler, we argue that the gender/sex binary is a tool of the patriarchy and thus any attempt to disrupt it also poses a danger to men’s power and status. Although this may happen in a number of ways, C. T. Nagoshi and colleagues (2019) argue that men’s status is particularly threatened by any indication that men, as a group, could be feminized. The authors propose that this concern is particularly pronounced in response to trans women. More specifically, the authors propose that feminization of men is associated with a loss of status and that if some men can be feminized (which is how they may view trans women), all men could potentially be feminized. This could in turn lead to a change in the social order (i.e., the patriarchy) such that men would no longer have higher status and more power than women. Trans women thus pose a threat not only to men’s individual manhood and status but also to their status more broadly. To a lesser extent, the same concerns are evoked by gay men, who are also seen as more feminine and thus threaten men’s status (see Warriner, Nagoshi, & Nagoshi, 2013). Again, this type of threat might be particularly pronounced for men who highly identify with their gender/sex but also for men who value hierarchy and the status quo, such as men with high scores on measures of right-wing authoritarianism or social-dominance orientation. In line with this argument, these constructs are related to higher levels of prejudice against trans people (Makwana et al., 2018).

Switching costumes (i.e., appearance-based gender trouble), which might also evoke status threat in men—and drag queens are an interesting example of gender trouble evoking group-based status threat. Drag performers are entertainers who generally dress up as the opposite (in binary terms) gender, often portraying exaggerated femininity or masculinity for entertainment purposes. Again, the effects of switching costumes are likely to be different among different members of the audience. Like trans women, drag performers, particularly drag queens, may pose a threat to masculinity in the ways described above. Interestingly, however, this may particularly be the case for gay men rather than heterosexual men. Because drag queens are often gay men themselves and thus part of the in-group of gay men, the overt feminization inherent in drag performances might be particularly threatening to gay men as a group. In other words, it may evoke concerns that they confirm the stereotype that gay men are effeminate and not “real men” (Kite & Deaux, 1987) and make gay men appear more feminine in general, thereby threatening gay men’s already precarious status (C. T. Nagoshi et al., 2019). This process may be particularly pronounced for men who subscribe to more traditional notions of masculinity. For those for whom this threat occurs, it is likely to lead to negative reactions to drag performers. There is, to our knowledge, scant psychological research on this topic, but Bishop, Kiss, Morrison, Rushe, and Specht (2014) show that gay men who endorse hypermasculinity indeed view drag queens more negatively (see also Berkowitz & Belgrave, 2010).

#### System threats

In addition to personal and group-based threats, gender trouble also poses a potential threat to the system of our society as a whole, as described by system-justification theory (Jost & Banaji, 1994; see also Jost, 2018). Drawing on the feminist concept of “false consciousness” (Cunningham, 1987), system-justification theory posits that individuals engage in behaviors that defend existing social and political structures. This is the case even if these structures disadvantage and oppress individuals or their groups because it makes people feel better about the status quo. Existing systems further reduce feelings of uncertainty, insecurity, and threat and provide structures that help coordinate social relationships and create a sense of shared reality. The gender/sex binary is an example of such a system, and hence disruptions to the gender/sex binary are likely to evoke system threat (e.g., in terms of uncertainty about how to categorize individuals or threat to traditional gender relations) and, in turn, efforts to reinforce and protect the system. This can happen even among groups who are disadvantaged by the gender/sex binary such as women, sexual- and gender-minority individuals, and those who violate gender norms.

The extent to which audience members engage in these system justification strategies depends on a range of individual and contextual factors (for a review, see Friesen, Laurin, Shepherd, Gaucher, & Kay, 2019). For example, individuals engage in more system-justifying behaviors when the system is perceived as having been in place for a long time (e.g., Blanchar & Eidelman, 2013), when individuals feel powerless or dependent on the system (e.g., van der Toorn, Tyler, & Jost, 2011), and for individuals with low need for cognition, high death anxiety, and high need to share reality (Hennes, Nam, Stern, & Jost, 2012). The fact that those who are politically conservative generally score higher in these constructs than those who are liberal fits with findings demonstrating that many of the strategies described above are often more pronounced among conservatives.

We argue that another group that may be particularly likely to experience system threat in the context of gender/sex are women, particularly some feminists. Although it may seem counterintuitive that feminists would be motivated to defend the gender/sex binary (and, indeed, many of them are actively trying to dismantle it), some feminist philosophies are very much rooted in the belief that in the current patriarchal system, women are oppressed from birth by men because of their biological sex (i.e., sex assigned at birth; e.g., Greer, 1999). Moreover, within such a perspective, gender roles and gender identity are seen as the result of socialization (a perspective that could in itself be seen as a form of gender trouble) and thus should be abolished. Here, then, feminism is defined as the struggle of (biological) women (the oppressed) against men (the oppressors; e.g., see Greer, 1999; Jeffreys, 2014). Clear, biologically based boundaries between the oppressor and the oppressed are thus at the core of this conceptualization of the feminist struggle—and on this basis, the blurring of these boundaries can be seen as problematic for the feminist cause. This is the case with trans women, who are seen as oppressors trying to enter the group of the oppressed, potentially undermining efforts to overthrow the patriarchal system. Although we know of no studies that have investigated the system-justifying motives of such feminist subgroups, these processes are illustrated by their strong and vocal opposition to trans-friendly policies and practices. For example, there has been some backlash from feminist subgroups in response to proposed changes to the UK’s Gender Recognition Act, which would make it easier for trans people to have their gender legally recognized and called for a third gender/sex option for nonbinary people on legal documents, a form of multigendering (Slawson, 2018). Such reactions, it should be noted, would be expected only among feminists who hold essentialist views of gender/sex (particularly in terms of innateness and immutability) and not among feminists who do not hold such beliefs.

System justification can take many different forms. Most relevant to the maintenance of the gender/sex binary are findings that demonstrate that system threat is associated with (a) selective and biased information processing to reach conclusions that support the system (E. L. Haines & Jost, 2000); (b) stereotyping of disadvantaged groups (e.g., women) as communal but not agentic and advantaged groups (e.g., men) as agentic but not communal (e.g., Laurin et al., 2011); (c) backlash against those who violate these stereotypes (e.g., agentic women; Rudman, Moss-Racusin, Glick, & Phelan, 2012) or who dare to openly challenge the system (e.g., feminists; Yeung, Kay, & Peach, 2014); (d) increased gender/sex essentialism (Brescoll et al., 2013); and (e) decreased support for collective action, for example, on behalf of women (Becker & Wright, 2011).

Gender trouble can cause system threat in a variety of ways. This includes deviating from the script, that is, behaving in a way that is not in line with gender stereotypes and norms. This is probably the most common form of gender trouble, illustrated, for example, by the increased representation of women in traditionally masculine roles (e.g., in leadership and science). In line with predictions from system-justification theory, any deviations from the script are likely to be penalized, particularly when they elicit system threat or when they directly threaten the status quo (Rudman, Moss-Racusin, Phelan, & Nauts, 2012). This backlash faced by gender troublemakers includes economic and social penalties for those deviating from the script, thus reinforcing gender norms and stereotypes—and hence the gender/sex binary (for a review, see Rudman, Moss-Racusin, Glick, & Phelan, 2012).

We argue that attempts to alleviate system threat can also take other forms that are less directly aimed at the troublemaker. First, research demonstrates that individuals who violate group stereotypes, such as gender troublemakers, are often subtyped as a way of keeping the stereotype intact (Kunda & Oleson, 1995). In other words, counterstereotypical group members, such as girls who like sports, are seen as exceptions to the rule and placed in their own category, here, tomboys, leaving the stereotypes (associated with girls and femininity) unchanged and contributing to their preservation. Gender troublemakers are thus likely to be subcategorized—for example as feminists, career women, gay men, or metrosexuals—allowing the generalized gender stereotypes to stay intact despite disconfirming evidence (Weber & Crocker, 1983).

Such subtyping can be seen as an attempt to realign character, costume, and script in the eyes of the audience. Because the script no longer matches the character and cannot easily be changed, changes to the character are made instead. For example, if a woman acts ambitiously and assertively at work, her character is changed from woman to career woman, which in turn comes with ideas about her costume that would be expected to be less feminine, for example, wearing a pant suit. Thus, although she is creating some gender trouble by deviating from her gender/sex script, the disruptive consequences for the gender/sex binary are minimized by keeping the character, costume, and script aligned. However, we would argue that the more extreme the deviation from the script, or the more widespread the behavior, the more disruptive to the gender/sex binary it becomes.

In a similar vein, although some behaviors can be seen as peripheral to what it means to be a man or a woman in our culture, others are more central and thus harbor the potential for more disruption. In many cultures, including Western culture, one of the most central aspects of gender/sex scripts, particularly for men, is heterosexuality (see Herek, 1986). Therefore, engaging in same-sex sexual behavior or displaying same-sex attraction is one of the most extreme form of deviating from gender/sex scripts and, because complementary, heterosexual relationships form an integral part of the gender/sex system, it is particularly likely to evoke system threat.

In line with Butler’s arguments, we propose that one way in which system threat can be reduced is again through the realignment of character, costume, and script. In the case of same-sex attraction and behavior, this can be accomplished by flipping the elements of character, costume, and other scripts. Although a career woman is still perceived as a woman, albeit a less feminine subtype of a woman, a gay man is seen as more woman than man and a lesbian woman as more man than woman—in other words, the character is changed to realign it with sexual behavior. To reinforce this idea, several authors have shown that stereotypes of gay men are often more similar to those of women than those of heterosexual men, whereas the opposite is true for lesbians (Brambilla, Carnaghi, & Ravenna, 2011; Clausell & Fiske, 2005; Vaughn, Teeters, Sadler, & Cronan, 2017). We suggest that this realignment also extends to the costume, with gay men being expected to adhere more to feminine beauty standards (e.g., removal of body hair, adherence to thinness ideals) and lesbians being expected to look more masculine (e.g., not wear makeup, have short hair, wear men’s clothing). We propose that when lesbians and gay men do not follow these prescriptions, other efforts will be made to preserve the gender/sex binary via the alignment of character, script, and costume. These efforts include the denial of identity (e.g., “It’s just a phase”; “You just haven’t met the right man yet”; see Blair & Hoskin, 2016; Levitt, Gerrish, & Hiestand, 2003) or the appropriation of lesbianism within a heterosexual context (e.g., pornography produced for a straight male audience; see Sender, 2004), which might be particularly pronounced for feminine lesbians. It also includes the reassignment of the previously denied, original character in a heteronormative fashion (e.g., “So you are the man/woman in the relationship”), particularly in situations in which one partner looks and acts more feminine/masculine, so that heteronormative ideals can be fulfilled (see K. M. Haines, Boyer, Giovanazzi, & Galupo, 2018). Other strategies for reducing the system threat caused by lesbian and gay identities may include opposition to LGB rights.

Although individuals who challenge the status quo are potentially threatening to the binary gender/sex system, attempts to dismantle the stage are likely to be even more problematic because they, by definition, aim to change the system itself. There is indirect evidence for this point demonstrating that conservatives (who are higher in system justification motives) strongly oppose attempts to dismantle the stage such as gender-neutral language (Gustafsson Sendén et al., 2015), same-sex marriage (Sherkat, Powell-Williams, Maddox, & De Vries, 2011), affirmative action to advance gender equality (Fraser, Osborne, & Sibley, 2015), and unisex bathrooms (Blumell et al., 2019).

**Fig. 3.**
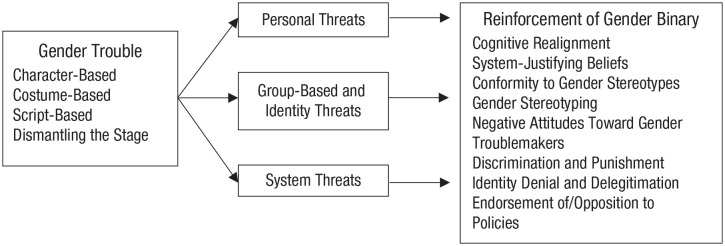
The psychological effects of gender trouble.

In summary, we have integrated different strands of the psychological literature into our proposed framework to help explain the psychological processes underlying the dynamics of the gender/sex binary and its potential disruption. Our framework thus integrates sociological, philosophical, and psychological theory and brings together disparate strands of the psychological literature that speak to the same issue. More specifically, we proposed that gender trouble can elicit personal threat, group-based and identity threat, and system threat and, in turn, elicit efforts to alleviate this threat and reinforce the gender/sex binary. We also argued that some forms of threats are particularly likely in response to specific forms of gender trouble and that some individuals and groups (e.g., men, conservatives, those who strongly identify with their gender, those with essentialist views, marginalized group members) are more likely to experience particular threats rather than others. Figure 3 gives an overview of these processes. From the literature we reviewed and integrated into our framework, it is clear that although different groups are more or less likely to experience different types of threat, the reactions to these threats are often indistinguishable from one another. This is in line with the observation that groups with very different values and aims—such as some conservatives and some radical feminists—may react in surprisingly similar ways, for example, to trans-friendly changes to policy and practice.

However, although it is important to understand the mechanisms involved in the perpetuation of the gender/sex binary and the potential resistance to change, we do not want to neglect the positive impact gender trouble can have. We turn to this issue next.

### The positive effects of gender trouble

We have described how gender trouble can lead to different forms of threat and, in turn, efforts to reduce the threat and reinforce the gender/sex binary. This may lead to the impression that gender trouble is at best useless and at worse harmful. However, we argue that this is not the case and that gender trouble can be the catalyst for social change—disrupting both the gender/sex binary and its harmful consequences. We can see many examples of this when looking at the changes to the gender/sex binary that have occurred in the past century and that were first met with strong opposition but are now accepted, at least by most, as completely normal such as women wearing trousers, women’s right to vote and to work, or coeducational schools.

Moreover, on the basis of the social-role theory (Eagly & Wood, 2012), we predict that, despite efforts to reinforce the gender/sex binary, widespread instances of gender trouble will, over time, lead to changes in gender stereotypes. For example, as more and more women enter the workforce and male-dominated fields, stereotypes of women are likely to include more aspects of agency (Diekman & Eagly, 2000), which in turn should give women more freedom to act in agentic ways and reduce the backlash to agentic women (see Bongiorno, Bain, & David, 2014). In line with this argument, recent evidence suggests that gender stereotypes have indeed changed—at least in some respects—over the past decades such that women are no longer seen as less competent than men (Eagly, Nater, Miller, Kaufmann, & Sczesny, 2020).

On a smaller scale, gender troublemakers can function as role models, showing others that they need not be restricted by the gender/sex binary and its prescriptions and proscriptions regarding character, costume, and script. Likewise, the increased visibility of those who play a different character (i.e., trans and nonbinary people), aided by the Internet, is likely to destabilize the link between sex and gender and increase the perceived fluidity of gender/sex. This should not only benefit those who feel as if their sex assigned at birth does not match their identity but also decrease gender/sex essentialism more generally. In line with this argument, the number of U.S. adults openly identifying as trans has doubled in the past 10 years (Flores et al., 2016). Even more strikingly, the number of nonbinary British university students has doubled from 2017 to 2018 (Higher Education Statistics Agency, 2018). We do not believe that this is a reflection of an increasing number of people who feel as if their gender/sex assigned at birth does not fit. Instead, we believe that it is a promising reflection of an increasing awareness and acceptance of nonbinary and trans identities that indicates that now, more than ever, trans and nonbinary individuals can feel free to publicly be themselves.

Last, recent changes suggest that attempts to dismantle the stage can be effective. Take for example the gender-neutral pronoun *hen* in the Swedish language. Like English and many other languages, Swedish has traditionally had two singular pronouns: *han* (he) and *hon* (she). However, in recent years, the gender-neutral word *hen* has gained popularity. *Hen* can be used to refer to a person whose gender is unknown as well as to nonbinary individuals. Although it was first suggested in 1966, it became well known outside of feminist and LGBTQ circles only in 2012, when a children’s book used only *hen* instead of *han* and *hon*, resulting in widespread media coverage and debate. Interestingly—and promisingly—since then, attitudes toward its use have shifted dramatically from predominantly negative to predominantly positive (Gustafsson Sendén et al., 2015). It seems to be viewed as truly gender-neutral. When participants in a recent study were asked to remember the gender/sex of a person whose gender/sex was not disclosed but who was described as either “the applicant” or as *hen*, most participants indicated that they had read about a man when they had read about “the applicant,” but this androcentric bias was not present for *hen* (Lindqvist et al., 2019).

Thus, both performance-based and context-based gender trouble can indeed lead to positive changes and, hopefully, over time, weaken the gender/sex binary. However, for these changes to be effective, there needs to be a cultural shift in how we view gender/sex. Although we would like to encourage everyone to free themselves of the restrictive prescriptions and proscriptions of the gender/sex binary, requiring everyone to become gender troublemakers is neither possible nor desirable. Individuals should be free to live and express their gender/sex authentically, in whatever way they see fit. Hence, our focus should be on the stage and how it can be set in a way that enables and highlights gender trouble and mitigates backlash against gender troublemakers.

## Summary and Conclusions

We have argued that neither sex nor gender is binary, that gender does not follow from sex, and that the gender/sex binary is harmful. We have extended Butler’s notion of gender performativity and Goffman’s metaphor of gender as a performance and argued that the performance of gender/sex includes the character one plays, the costume one wears, and the script one enacts. Gender trouble can be created by misaligning those three elements or by challenging their immutability. Moreover, gender/sex is performed on a stage. This stage can be dismantled by degendering or multigendering the context in ways that facilitate gender trouble. We have used this framework to integrate various pieces of the social-psychological literature and argued that gender trouble can evoke personal, group-based and identity, and system threat, and, in turn, efforts to eliminate the threat and reinforce the gender/sex binary. However, despite these hurdles, gender trouble can lead to social change and less binary, more inclusive conceptions of gender/sex.

The framework we have presented engenders many novel research questions that need to be answered empirically. We have proposed that the binary alignment of character, costume, and script reinforces the gender/sex binary and that adjustments are made when these three elements are not aligned. Although there is evidence for some of these ideas (e.g., that individuals use gender/sex to infer information about likely behaviors and appearances but also use information about appearance to predict behavior; Deaux & Lewis, 1984), there are novel predictions that have yet to be tested. For example, does alignment indeed reinforce the gender/sex binary, including views that there are only two sexes and two genders and that gender follows from sex? Does the misalignment of character, costume, and script indeed cause gender trouble?

Moreover, we have discussed several reasons why gender trouble may backfire and lead to attempts to reinforce the gender/sex binary. It is therefore worth exploring what kind of gender trouble is most effective in disrupting the gender/sex binary, for example, because it causes less threat. One of the open questions concerns whether permanent or temporary gender trouble is more effective. Moreover, we have highlighted that degendering might be particularly threatening—so is multigendering the way to go? Or will this strategy just lead to a new, third category in people’s mind without changing any of the prescriptions and proscriptions associated with men and women? On a similar note, it is important to investigate how threat can be diminished to make gender trouble more effective.

As we have demonstrated, there is an abundance of evidence in the psychological literature that speaks to the reactions to gender trouble, albeit not labeled as such. For example, many researchers have examined reactions to women and men who violate gender stereotypes (i.e., those who deviate from the script). However, other forms of gender trouble have received much less attention. We hope that this article will inspire research on less commonly researched types of gender trouble, for example, nonbinary identities or nonprototypical sexual-minority groups (e.g., feminine lesbian women or masculine gay men). In line with Butler, we also believe that examining reactions to drag performers is a valuable avenue to pursue. Building on Newton (1968), Butler discusses the power of drag queens in particular to subvert the gender/sex binary (and we would argue that the same applies to drag kings). In her anthropological work, Newton argues that the various layers of drag disrupt the gender/sex binary in multiple ways: Drag queens appear feminine on the outside, but the body on the inside is usually male. At the same time, the outside, that is, the body, is male, but the inside, that is, the “essence” (i.e., the performed gender, the character), of a drag queen is feminine, as illustrated by the fact that female pronouns are generally used when referring to drag queens. In addition, Butler argues that the exaggerated portrayal of femininity often exhibited by drag queens makes the performative nature of gender/sex we all engage in more visible. She argues that all gendered performance is drag in that it imitates an unrealistic, fabricated ideal of femininity and masculinity—but this is largely invisible in everyday life. Drag makes this process visible. We therefore argue that although studying drag performances may not seem particularly generalizable, it is an avenue worth pursuing as an opportunity for studying gender/sex in a setting that is less stable and more obviously performed than most other contexts.

In addition to new research questions, this article also highlights the need to step away from binary conceptions of gender/sex in psychological theorizing and research. To bring about the cultural change necessary to weaken the gender/sex binary and enable gender trouble, we, as psychologists, need to change the way in which we treat gender/sex (see Morgenroth & Ryan, 2018). For example, we should move away from our obsession with binary gender/sex differences and from viewing gender/sex as the independent variable that explains behavior. Such practices are not only inappropriate, given that gender/sex is not a categorical construct (see Carothers & Reis, 2013), but also part of the performative creation of gender. By assuming gender/sex as binary and natural and treating it as such in our designs and analyses (e.g., by removing nonbinary participants from our analyses and comparing women to men), we produce findings that reflect this dichotomy. Instead, we should treat gender/sex as an outcome of cultural, psychological, and behavioral processes—and this needs to be reflected both in our theorizing and our practices (e.g., how we measure gender). For example, given our arguments above, it is important to decide which aspect of gender/sex is relevant for a specific research question (i.e., the character, costume, or script) and to treat it as a state-like rather than trait-like characteristic and as dimensional rather than categorical, reflecting the fact that character, costume, and script are not necessarily stable and clear-cut. An example of such an approach is the work by van Anders and colleagues (Abed, Schudson, Gunther, Beischel, & van Anders, 2019; Schudson, Manley, Diamond, & van Anders, 2018; van Anders, 2015), who use sexual-configurations theory as a framework for studying gender/sex and sexuality. More specifically, this approach uses diagrams in which participants can separately indicate different aspects of their gender/sex such as masculinity/femininity, gender identity, and sex, as well as the significance or strength of these gendered/sexed aspects. It is important to note that this approach has been successfully used with both gender- and sexual-minority groups (Schudson et al., 2018) as well as cisgender participants (Abed et al., 2019).

Although we have primarily drawn from the social-psychological literature, our arguments have important implications for anyone studying gender. For example, personality psychologists studying gender/sex differences in personality traits may want to reconsider whether these traits are indeed the result of gender/sex or part of its socially sanctioned performance; biological psychologists may want to pay more attention to the role that biology plays in the performance of gender/sex—and the role that the performance of gender/sex plays in sex differences; and last, psychologists studying or interacting with clinical populations may use the recommendations above to treat gender/sex in a more inclusive and less stigmatizing way. In other words, all psychologists should critically reflect on how their field maintains and reinforces the gender/sex binary. For example, testing for binary gender/sex differences, particularly when there are no a priori hypotheses regarding these differences, will result in Type I errors and reinforce the belief that there are two genders/sexes with meaningful differences. Likewise, pathologizing different forms of gender trouble signals that some ways of performing gender are “right” whereas others are “wrong.” For example, although the fifth edition of the *Diagnostic and Statistical Manual of Mental Disorders* (American Psychiatric Association, 2013) no longer contains “gender identity disorder” as a sexual disorder and has instead replaced it with “gender dysphoria,” the 10th edition of the *International Classification of Diseases* (World Health Organization, 2016) still lists “transsexualism,” “dual-role transvestism,” and “gender identity disorder” as disorders (although it should be noted that this will no longer be the case in the 11th edition, which is scheduled be published in 2022).

To conclude, we have argued that many gendered constructs and processes that are examined in psychology (e.g., transphobia, backlash against agentic women, precarious manhood) are part of one system—the gender/sex binary—and dismantling this system will benefit a wide range of people. One may wonder, however, how realistic this goal really is. Categorizing (e.g., into male and female) is a useful heuristic, and it is hard to imagine that we could function effectively without it. Others might argue that gender/sex categories provide an important sense of solidarity that can be used to encourage collective action and create a more equal society and an authentic feeling of identity. To be clear, we are not suggesting that getting rid of the categories women and men should be everyone’s goals. Instead, gender trouble can help break the shackles of the gender/sex binary, expand our notions of gender/sex, and enable *everyone* to live their gender/sex authentically and without fear of repercussions.

## Transparency

*Action Editor:* Laura A. King

*Editor:* Laura A. King

## Notes

1. It should be noted that Butler has repeatedly distanced herself from Goffman’s work, particularly from the idea of an active “performer” or “actor.” Goffman sees the gendered self as something created through the performance of gender. Butler rejects this idea and, instead, argues that the illusion of a gendered self is created through the performance of gender/sex and that, ultimately, there is no “self.” Both agree, however, that there is no gendered self or identity—whether illusionary or real—before the performance of gender and that cultural and social processes regulate the performance of gender. They also both reject the distinction between sex and gender and the existence of prediscursive sexed bodies. Overall, we would argue that although the distinctions and incompatibilities between Butler and Goffman are interesting and important from a philosophical point of view, they are less important for the purpose of this article. Indeed, we believe that the two approaches complement one another and together provide a good foundation for a theoretical framework for understanding the perpetuation and disruption of the gender/sex binary.

## References

[bibr1-1745691620902442] Abed E. C. Schudson Z. C. Gunther O. D. Beischel W. J. van Anders S. M. (2019). Sexual and gender diversity among sexual and gender/sex majorities: Insights via sexual configurations theory. Archives of Sexual Behavior, 48, 1423–1442. doi:10.1007/s10508-018-1340-2 31123948

[bibr2-1745691620902442] Ainsworth C. (2015). Sex redefined. Nature News, 518, 288–291. doi:10.1038/518288a 25693544

[bibr3-1745691620902442] American Psychiatric Association. (2013). Diagnostic and statistical manual of mental disorders (5th ed.). doi:10.1176/appi.books.9780890425596

[bibr4-1745691620902442] American Psychological Association. (2018). APA guidelines for psychological practice with boys and men. Retrieved from http://www.apa.org/about/policy/psychological-practice-boys-men-guidelines.pdf

[bibr5-1745691620902442] Ansara Y. G. Hegarty P. (2014). Methodologies of misgendering: Recommendations for reducing cisgenderism in psychological research. Feminism & Psychology, 24, 259–270. doi:10.1177/0959353514526217

[bibr6-1745691620902442] Arboleda V. A. Sandberg D. E. Vilain E. (2014). DSDs: Genetics, underlying pathologies and psychosexual differentiation. Nature Reviews Endocrinology, 10, 603–615. doi:10.1038/nrendo.2014.130 PMC4441533 25091731

[bibr7-1745691620902442] Austin J. L. (1962). How to do things with words. Oxford, England: Clarendon Press.

[bibr8-1745691620902442] Bailey J. M. Vasey P. L. Diamond L. M. Breedlove S. M. Vilain E. Epprecht M. (2016). Sexual orientation, controversy, and science. Psychological Science in the Public Interest, 17, 45–101. 27113562 10.1177/1529100616637616

[bibr9-1745691620902442] Barreto M. Ellemers N. (2005). The burden of benevolent sexism: How it contributes to the maintenance of gender inequalities. European Journal of Social Psychology, 35, 633–642. doi:10.1002/ejsp.270

[bibr10-1745691620902442] Becker J. C. Wright S. C. (2011). Yet another dark side of chivalry: Benevolent sexism undermines and hostile sexism motivates collective action for social change. Journal of Personality and Social Psychology, 101, 62–77. doi:10.1037/a0022615 21355657

[bibr11-1745691620902442] Bem S. L. (1995). Dismantling gender polarization and compulsory heterosexuality: Should we turn the volume down or up? Journal of Sex Research, 32, 329–334. doi:10.1080/00224499509551806

[bibr12-1745691620902442] Berkowitz D. Belgrave L. (2010). “She works hard for the money”: Drag queens and the management of their contradictory status of celebrity and marginality. Journal of Contemporary Ethnography, 39, 159–186. doi:10.1177/0891241609342193

[bibr13-1745691620902442] Bishop C. J. Kiss M. Morrison T. G. Rushe D. M. Specht J. (2014). The association between gay men’s stereotypic beliefs about drag queens and their endorsement of hypermasculinity. Journal of Homosexuality, 61, 554–567. doi:10.1080/00918369.2014.865464 24245592

[bibr14-1745691620902442] Blair K. L. Hoskin R. A. (2016). Contemporary understandings of femme identities and related experiences of discrimination. Psychology & Sexuality, 7, 101–115. doi:10.1080/19419899.2015.1053824

[bibr15-1745691620902442] Blanchar J. C. Eidelman S. (2013). Perceived system longevity increases system justification and the legitimacy of inequality. European Journal of Social Psychology, 43, 238–245. doi:10.1002/ejsp.1960

[bibr16-1745691620902442] Blumell L. E. Huemmer J. Sternadori M. (2019). Protecting the ladies: Benevolent sexism, heteronormativity, and partisanship in online discussions of gender-neutral bathrooms. Mass Communication and Society, 22, 365–388. doi:10.1080/15205436.2018.1547833

[bibr17-1745691620902442] Bongiorno R. Bain P. G. David B. (2014). If you’re going to be a leader, at least act like it! Prejudice towards women who are tentative in leader roles. British Journal of Social Psychology, 53, 217–234. doi:10.1111/bjso.12032 23509967

[bibr18-1745691620902442] Bosson J. K. Vandello J. A. Caswell T. A. (2013). Precarious manhood. In Ryan M. K. Branscombe N. R. (Eds.), The SAGE handbook of gender and psychology (pp. 15–130). Thousand Oaks, CA: SAGE.

[bibr19-1745691620902442] Boylan J. F. (2018, January 9). That’s what ze said. The New York Times. Retrieved from https://www.nytimes.com

[bibr20-1745691620902442] Brambilla M. Carnaghi A. Ravenna M. (2011). Status and cooperation shape lesbian stereotypes: Testing predictions from the stereotype content model. Social Psychology, 42, 101–110. doi:10.1027/1864-9335/a000054

[bibr21-1745691620902442] Branscombe N. R. Ellemers N. Spears R. Doosje B. (1999). The context and content of social identity threat. In Ellemers N. Spears R. Doosje B. (Eds.), Social identity: Context, commitment, content (pp. 35–59). Oxford, England: Blackwell.

[bibr22-1745691620902442] Brescoll V. L. Uhlmann E. L. Newman G. E. (2013). The effects of system-justifying motives on endorsement of essentialist explanations for gender differences. Journal of Personality and Social Psychology, 105, 891–908. doi:10.1037/a0034701 24295379

[bibr23-1745691620902442] Brewster M. E. Moradi B. (2010). Perceived experiences of anti-bisexual prejudice: Instrument development and evaluation. Journal of Counseling Psychology, 57, 451–468. doi:10.1037/a0021116 27078194

[bibr24-1745691620902442] Burke S. E. LaFrance M. (2018). Perceptions of instability and choice across sexual orientation groups. Group Processes & Intergroup Relations, 21, 257–279. doi:10.1177/1368430216663019

[bibr25-1745691620902442] Butler J. (1990). Gender trouble: Feminism and the subversion of identity. London, England: Routledge.

[bibr26-1745691620902442] Carothers B. J. Reis H. T. (2013). Men and women are from Earth: Examining the latent structure of gender. Journal of Personality and Social Psychology, 104, 385–407. doi:10.1037/a0030437 23088230

[bibr27-1745691620902442] Clausell E. Fiske S. T. (2005). When do subgroup parts add up to the stereotypic whole? Mixed stereotype content for gay male subgroups explains overall ratings. Social Cognition, 23, 161–181. doi:10.1521/soco.23.2.161.65626

[bibr28-1745691620902442] Coleman J. M. Hong Y. Y. (2008). Beyond nature and nurture: The influence of lay gender theories on self-stereotyping. Self and Identity, 7, 34–53. doi:10.1080/15298860600980185

[bibr29-1745691620902442] Collins P. H. (2004). Black sexual politics: African Americans, gender, and the new racism. New York, NY: Routledge. 10.1080/13691058.2013.872316 24455983

[bibr30-1745691620902442] Cunningham F. (1987). False consciousness. In Cunningham F. (Ed.), Democratic theory and socialism (pp. 236–267). Cambridge, England: Cambridge University Press.

[bibr31-1745691620902442] Deaux K. (1991). Social identities: Thoughts on structure and change. In Curtis R. C. (Ed.), The relational self: Theoretical convergences in psychoanalysis and social psychology (pp. 77–93). New York, NY: Guilford Press.

[bibr32-1745691620902442] Deaux K. Lewis L. L. (1984). Structure of gender stereotypes: Interrelationships among components and gender label. Journal of Personality and Social Psychology, 46, 991–1004. doi:10.1037/0022-3514.46.5.991

[bibr33-1745691620902442] Deaux K. Major B. (1987). Putting gender into context: An interactive model of gender-related behavior. Psychological Review, 94, 369–389. doi:10.1037/0033-295X.94.3.369

[bibr34-1745691620902442] DeSouza E. R. Wesselmann E. D. Ispas D. (2017). Workplace discrimination against sexual minorities: Subtle and not-so-subtle. Canadian Journal of Administrative Sciences/Revue Canadienne des Sciences de l’Administration, 34, 121–132. doi:10.1002/cjas.1438

[bibr35-1745691620902442] Devor H. (1997). More than manly women: How female-to-male transsexuals reject lesbian identities. In Bullough B. Bullough V. Elias J. (Eds.), Gender blending (pp. 87–102). Amherst, NY: Prometheus.

[bibr36-1745691620902442] Diekman A. B. Eagly A. H. (2000). Stereotypes as dynamic constructs: Women and men of the past, present, and future. Personality and Social Psychology Bulletin, 26, 1171–1188. doi:10.1177/0146167200262001

[bibr37-1745691620902442] Donovan R. A. (2011). Tough or tender: (Dis)similarities in White college students’ perceptions of Black and White women. Psychology of Women Quarterly, 35, 458–468. doi:10.1177/0361684311406874

[bibr38-1745691620902442] Drover M. (2017, March 26). Fashion crimes: The rabbit hole of criminalized cross-dressing in US. Retrieved from http://co-op.antiochcollege.edu/fashion-crimes-the-rabbit-hole-of-criminalized-cross-dressing-in-us-history

[bibr39-1745691620902442] Dyar C. London B. (2018). Longitudinal examination of a bisexual-specific minority stress process among bisexual cisgender women. Psychology of Women Quarterly, 42, 342–360. doi:10.1177/0361684318768233

[bibr40-1745691620902442] Eagly A. H. (1987). Sex differences in social behavior: A social-role interpretation. Hillsdale, NJ: Erlbaum.

[bibr41-1745691620902442] Eagly A. H. Nater C. Miller D. I. Kaufmann M. Sczesny S. (2020). Gender stereotypes have changed: A cross-temporal meta-analysis of U.S. public opinion polls from 1946 to 2018. American Psychologist, 75(3), 301–315. doi:10.1037/amp0000494 31318237

[bibr42-1745691620902442] Eagly A. H. Wood W. (2012). Social role theory. In Van Lange P. A. M. Kruglanski A. W. Higgins E. T. (Eds.), Handbook of theories of social psychology (pp. 458–476). Thousand Oaks, CA: SAGE.

[bibr43-1745691620902442] Earles J. (2019). The “penis police”: Lesbian and feminist spaces, trans women, and the maintenance of the sex/gender/sexuality system. Journal of Lesbian Studies, 23, 243–256. doi:10.1080/10894160.2018.1517574 30472929

[bibr44-1745691620902442] Eddy M. Bennett J. (2017, November 8). Germany must allow third gender category, court rules. The New York Times. Retrieved from https://www.nytimes.com

[bibr45-1745691620902442] Eliason M. J. (1997). The prevalence and nature of biphobia in heterosexual undergraduate students. Archives of Sexual Behavior, 26, 317–326. doi:10.1023/A:1024527032040 9146816

[bibr46-1745691620902442] Falomir-Pichastor J. M. Hegarty P. (2014). Maintaining distinctions under threat: Heterosexual men endorse the biological theory of sexuality when equality is the norm. British Journal of Social Psychology, 53, 731–751. doi:10.1111/bjso.12051 24131397

[bibr47-1745691620902442] Fausto-Sterling A. (1993). The five sexes: Why male and female are not enough. The Sciences, 33, 19–24.

[bibr48-1745691620902442] Fausto-Sterling A. (2000). Sexing the body: Gender politics and the construction of sexuality (1st ed.). New York, NY: Basic Books.

[bibr49-1745691620902442] Fausto-Sterling A. (2019). Gender/sex, sexual orientation, and identity are in the body: How did they get there? Journal of Sex Research, 56, 529–555. doi:10.1080/00224499.2019.1581883 30875248

[bibr50-1745691620902442] Federal Bureau of Prisons. (2019, September 7). Inmate gender. Retrieved from https://www.bop.gov/about/statistics/statistics_inmate_gender.jsp

[bibr51-1745691620902442] Fine C. (2010). Delusions of gender: How our minds, society, and neurosexism create difference. New York, NY: W.W. Norton.

[bibr52-1745691620902442] Fine C. (2017). Testosterone Rex: Myths of sex, science, and society. New York, NY: W. W. Norton.

[bibr53-1745691620902442] Fiske S. T. (2010). Venus and Mars or down to Earth: Stereotypes and realities of gender differences. Perspectives on Psychological Science, 5, 688–692. doi:10.1177/1745691610388768 23678365 PMC3652639

[bibr54-1745691620902442] Fiske S. T. Cuddy A. Glick P. Xu J. (2002). A model of (often mixed) stereotype content: Competence and warmth respectively follow from perceived status and competition. Journal of Personality and Social Psychology, 82, 878–902. doi:10.1037//0022-3514.82.6.878 12051578

[bibr55-1745691620902442] Flores A. R. Herman J. L. Gates G. J. Brown T. N. T. (2016). How many adults identify as transgender in the United States? Los Angeles, CA: The Williams Institute.

[bibr56-1745691620902442] Fraser G. Osborne D. Sibley C. G. (2015). “We want you in the workplace, but only in a skirt!” Social dominance orientation, gender-based affirmative action and the moderating role of benevolent sexism. Sex Roles, 73, 231–244. doi:10.1007/s11199-015-0515-8

[bibr57-1745691620902442] Friedman E. J. (2014). Cisgenderism in gender attributions: The ways in which social, cognitive, and individual factors predict misgendering (Doctoral dissertation). The City University of New York, New York.

[bibr58-1745691620902442] Friesen J. P. Laurin K. Shepherd S. Gaucher D. Kay A. C. (2019). System justification: Experimental evidence, its contextual nature, and implications for social change. British Journal of Social Psychology, 58, 315–339. doi:10.1111/bjso.12278 30229936

[bibr59-1745691620902442] Fyock J. Stangor C. (1994). The role of memory biases in stereotype maintenance. British Journal of Social Psychology, 33, 331–343. doi:10.1111/j.2044-8309.1994.tb01029.x 7953221

[bibr60-1745691620902442] Garelick A. S. Filip-Crawford G. Varley A. H. Nagoshi C. T. Nagoshi J. L. Evans R. (2017). Beyond the binary: Exploring the role of ambiguity in biphobia and transphobia. Journal of Bisexuality, 17, 172–189. doi:10.1080/15299716.2017.1319890

[bibr61-1745691620902442] Gender Identity Development Service. (2019). Referrals to GIDS, 2014-15 to 2018-19. Retrieved from http://gids.nhs.uk/number-referrals

[bibr62-1745691620902442] Ghavami N. Peplau L. A. (2012). An intersectional analysis of gender and ethnic stereotypes. Psychology of Women Quarterly, 37, 113–127. doi:10.1177/0361684312464203

[bibr63-1745691620902442] Glen F. Hurrell K. (2012). Technical note: Measuring gender identity. Equality and Human Rights Commission. Retrieved from https://www.equalityhumanrights.com/sites/default/files/technical_note_final.pdf

[bibr64-1745691620902442] Glick P. Fiske S. T. (1996). The ambivalent sexism inventory: Differentiating hostile and benevolent sexism. Journal of Personality and Social Psychology, 70, 491–512. doi:10.1037/0022-3514.70.3.491

[bibr65-1745691620902442] Glick P. Fiske S. T. (2001). Ambivalent sexism. In Advances in experimental social psychology (Vol. 33, pp. 115–188). San Diego, CA: Academic Press.

[bibr66-1745691620902442] Goffman E. (1959). The presentation of self in everyday life. Garden City, NY: Doubleday.

[bibr67-1745691620902442] Graves D. A. (2007). The experience of being a “bear”: A phenomenological study of an American gay subculture. Dissertation Abstracts International, 68, 1927–2232.

[bibr68-1745691620902442] Greer G. (1999). The whole woman. London, England: Knopf Doubleday Publishing Group.

[bibr69-1745691620902442] Grierson J. (2017, November 8). Virginia elects transgender woman to state legislature. The Guardian. Retrieved from https://www.theguardian.com

[bibr70-1745691620902442] Gustafsson Sendén M. Bäck E. A. Lindqvist A. (2015). Introducing a gender-neutral pronoun in a natural gender language: The influence of time on attitudes and behavior. Frontiers in Psychology, 6, Article 893. doi:10.3389/fpsyg.2015.00893 PMC4486751 26191016

[bibr71-1745691620902442] Haines E. L. Deaux K. Lofaro N. (2016). The times they are a-changing . . . or are they not? A comparison of gender stereotypes, 1983–2014. Psychology of Women Quarterly, 40, 353–363. doi:10.1177/0361684316634081

[bibr72-1745691620902442] Haines E. L. Jost J. T. (2000). Placating the powerless: Effects of legitimate and illegitimate explanation on affect, memory, and stereotyping. Social Justice Research, 13, 219–236. doi:10.1023/A:1026481205719

[bibr73-1745691620902442] Haines K. M. Boyer C. R. Giovanazzi C. Galupo M. P. (2018). “Not a real family”: Microaggressions directed toward LGBTQ families. Journal of Homosexuality, 65, 1138–1151. doi:10.1080/00918369.2017.1406217 29144852

[bibr74-1745691620902442] Haslam N. Rothschild L. Ernst D. (2000). Essentialist beliefs about social categories. British Journal of Social Psychology, 39, 113–127. doi:10.1348/014466600164363 10774531

[bibr75-1745691620902442] Helms J. L. Waters A. M. (2016). Attitudes toward bisexual men and women. Journal of Bisexuality, 16, 454–467. doi:10.1080/15299716.2016.1242104

[bibr76-1745691620902442] Hennes E. P. Nam H. H. Stern C. Jost J. T. (2012). Not all ideologies are created equal: Epistemic, existential, and relational needs predict system-justifying attitudes. Social Cognition, 30, 669–688. doi:10.1521/soco.2012.30.6.669

[bibr77-1745691620902442] Herdt G. (Ed.). (1993). Third sex, third gender: Beyond sexual dimorphism in culture and history. New York, NY: Zone Books.

[bibr78-1745691620902442] Herek G. M. (1986). On heterosexual masculinity: Some psychical consequences of the social construction of gender and sexuality. In Kimmel M. S. (Ed.), Sage focus editions, Vol. 88. Changing men: New directions in research on men and masculinity (pp. 68–82). Thousand Oaks, CA: SAGE.

[bibr79-1745691620902442] Herek G. M. (2002a). Gender gaps in public opinion about lesbians and gay men. Public Opinion Quarterly, 66, 40–66.

[bibr80-1745691620902442] Herek G. M. (2002b). Heterosexuals’ attitudes toward bisexual men and women in the United States. Journal of Sex Research, 39, 264–274. doi:10.1080/00224490209552150 12545409

[bibr81-1745691620902442] Higher Education Statistics Agency. (2018). Who’s studying in HE? Retrieved from https://www.hesa.ac.uk/data-and-analysis/students/whos-in-he

[bibr82-1745691620902442] Hill D. B. Willoughby B. L. (2005). The development and validation of the genderism and transphobia scale. Sex Roles, 53, 531–544. doi:10.1007/s11199-005-7140-x

[bibr83-1745691620902442] Hines S. (2019). The feminist frontier: On trans and feminism. Journal of Gender Studies, 28, 145–157. doi:10.1080/09589236.2017.1411791

[bibr84-1745691620902442] Hornsey M. J. Jetten J. (2003). Not being what you claim to be: Impostors as sources of group threat. European Journal of Social Psychology, 33, 639–657. doi:10.1002/ejsp.176

[bibr85-1745691620902442] Howansky K. Wilton L. S. Young D. M. Abrams S. Clapham R. (2021). (Trans)gender stereotypes and the self: Content and consequences of gender identity stereotypes. Self and Identity, 20(4), 478–495. 10.1080/15298868.2019.1617191

[bibr86-1745691620902442] Hoyt C. L. Morgenroth T. Burnette J. L. (2019). Understanding sexual prejudice: The role of political ideology and strategic essentialism. Journal of Applied Social Psychology, 49(1), 3–14. doi:10.1111/jasp.12560

[bibr87-1745691620902442] Human Rights Campaign. (2018, November 19). A national epidemic: Fatal anti-transgender violence in America in 2018. Retrieved from https://assets2.hrc.org/files/assets/resources/AntiTransViolence-2018Report-Final.pdf?_ga=2.87107992.1087835002.1549390089-45596882.1549390089

[bibr88-1745691620902442] Hyde J. S. (2005). The gender similarities hypothesis. American Psychologist, 60, 581–592. doi:10.1037/0003-066X.60.6.581 16173891

[bibr89-1745691620902442] Hyde J. S. Bigler R. S. Joel D. Tate C. C. van Anders S. M. (2019). The future of sex and gender in psychology: Five challenges to the gender binary. American Psychologist, 74, 171–193. doi:10.1037/amp0000307 30024214

[bibr90-1745691620902442] Jeffreys S. (2014). Gender hurts: A feminist analysis of the politics of transgenderism. Abingdon, England: Routledge.

[bibr91-1745691620902442] Joel D. Berman Z. Tavor I. Wexler N. Gaber O. Stein Y. Liem F. (2015). Sex beyond the genitalia: The human brain mosaic. Proceedings of the National Academy of Sciences, USA, 112, 15468–15473. doi:10.1073/pnas.1509654112 PMC4687544 26621705

[bibr92-1745691620902442] Joel D. Tarrasch R. Berman Z. Mukamel M. Ziv E. (2014). Queering gender: Studying gender identity in ‘normative’ individuals. Psychology & Sexuality, 5, 291–321. doi:10.1080/19419899.2013.830640

[bibr93-1745691620902442] Jost J. T. (2018). A quarter century of system justification theory: Questions, answers, criticisms, and societal applications. British Journal of Social Psychology, 58, 263–314. doi:10.1111/bjso.12297

[bibr94-1745691620902442] Jost J. T. Banaji M. R. (1994). The role of stereotyping in system-justification and the production of false consciousness. British Journal of Social Psychology, 33, 1–27. doi:10.1111/j.2044-8309.1994.tb01008.x

[bibr95-1745691620902442] Katz-Wise S. L. Budge S. L. Fugate E. Flanagan K. Touloumtzis C. Rood B. Leibowitz S. (2017). Transactional pathways of transgender identity development in transgender and gender-nonconforming youth and caregiver perspectives from the trans youth family study. International Journal of Transgenderism, 18, 243–263. doi:10.1080/15532739.2017.1304312 PMC5844490 29527139

[bibr96-1745691620902442] Katz-Wise S. L. Hyde J. S. (2012). Victimization experiences of lesbian, gay, and bisexual individuals: A meta-analysis. Journal of Sex Research, 49, 142–167. doi:10.1080/00224499.2011.637247 22380586

[bibr97-1745691620902442] Kite M. E. Deaux K. (1987). Gender belief systems: Homosexuality and the implicit inversion theory. Psychology of Women Quarterly, 11, 83–96. doi:10.1111/j.1471-6402.1987.tb00776.x

[bibr98-1745691620902442] Korolczuk E. Graff A. (2018). Gender as “Ebola from Brussels”: The anti-colonial frame and the rise of illiberal populism. Signs: Journal of Women in Culture and Society, 43, 797–821. doi:10.1086/696691

[bibr99-1745691620902442] Kroeper K. M. Sanchez D. T. Himmelstein M. S. (2014). Heterosexual men’s confrontation of sexual prejudice: The role of precarious manhood. Sex Roles, 70, 1–13. doi:10.1007/s11199-013-0306-z

[bibr100-1745691620902442] Kunda Z. Oleson K. C. (1995). Maintaining stereotypes in the face of disconfirmation: Constructing grounds for subtyping deviants. Journal of Personality and Social Psychology, 68, 565–579. doi:10.1037/0022-3514.68.4.565 7738766

[bibr101-1745691620902442] Landrine H. (1985). Race × class stereotypes of women. Sex Roles, 13, 65–75.

[bibr102-1745691620902442] Latrofa M. Vaes J. Cadinu M. Carnaghi A. (2010). The cognitive representation of self-stereotyping. Personality and Social Psychology Bulletin, 36, 911–922. doi:10.1177/0146167210373907 20519574

[bibr103-1745691620902442] Laurin K. Kay A. C. Shepherd S. (2011). Self-stereotyping as a route to system justification. Social Cognition, 29, 360–375. doi:10.1521/soco.2011.29.3.360

[bibr104-1745691620902442] Lee H. K. (2003, February 25). Guilty plea in transgender killing: Defendant makes deal, testifies against friends. San Francisco Chronicle. Retrieved from https://www.sfgate.com

[bibr105-1745691620902442] Levant R. F. Richmond K. (2007). A review of research on masculinity ideologies using the Male Role Norms Inventory. The Journal of Men’s Studies, 15, 130–146.

[bibr106-1745691620902442] Levitt H. M. (2019). A psychosocial genealogy of LGBTQ+ gender: An empirically based theory of gender and gender identity cultures. Psychology of Women Quarterly, 43, 275–297. doi:10.1177/0361684319834641

[bibr107-1745691620902442] Levitt H. M. Gerrish E. A. Hiestand K. R. (2003). The misunderstood gender: A model of modern femme identity. Sex Roles, 48, 99–113. doi:10.1023/A:1022453304384

[bibr108-1745691620902442] Levitt H. M. Horne S. G. (2002). Explorations of lesbian-queer genders. Journal of Lesbian Studies, 6, 25–39. doi:10.1300/J155v06n02_05 24807656

[bibr109-1745691620902442] Levitt H. M. Puckett J. A. Ippolito M. R. Horne S. G. (2012). Sexual minority women’s gender identity and expression: Challenges and supports. Journal of Lesbian Studies, 16, 153–176. doi:10.1080/10894160.2011.605009 22455340

[bibr110-1745691620902442] LGBT Foundation. (2017). Non-binary inclusion. Retrieved from https://lgbt.foundation/who-we-help/trans-people/non-binary

[bibr111-1745691620902442] Liening S. H. Stanton S. J. Saini E. K. Schultheiss O. C. (2010). Salivary testosterone, cortisol, and progesterone: Two-week stability, interhormone correlations, and effects of time of day, menstrual cycle, and oral contraceptive use on steroid hormone levels. Physiology & Behavior, 99, 8–16. doi:10.1016/j.physbeh.2009.10.001 19833145

[bibr112-1745691620902442] Lindqvist A. Renström E. A. Gustafsson Sendén M. (2019). Reducing a male bias in language? Establishing the efficiency of three different gender-fair language strategies. Sex Roles, 81, 109–117. doi:10.1007/s11199-018-0974-9

[bibr113-1745691620902442] Lippa R. A. (2010). Gender differences in personality and interests: When, where, and why? Social & Personality Psychology Compass, 4, 1098–1110. doi:10.1111/j.1751-9004.2010.00320.x

[bibr114-1745691620902442] Magnusson K. (2014, January 13). Interpreting Cohen’s d effect size. Retrieved from https://rpsychologist.com/d3/cohend

[bibr115-1745691620902442] Majors R. G. Billson J. M. (1992). Cool pose: The dilemmas of Black manhood in America. New York, NY: Lexington Books.

[bibr116-1745691620902442] Makwana A. P. Dhont K. Akhlaghi-Ghaffarokh P. Masure M. Roets A. (2018). The motivated cognitive basis of transphobia: The roles of right-wing ideologies and gender role beliefs. Sex Roles, 79, 206–217. doi:10.1007/s11199-017-0860-x

[bibr117-1745691620902442] Mann S. L. (2011). Drag queens’ use of language and the performance of blurred gendered and racial identities. Journal of Homosexuality, 58, 793–811. doi:10.1080/00918369.2011.581923 21740211

[bibr118-1745691620902442] Marques J. M. Yzerbyt V. Y. Leyens J.-P. (1988). The “Black Sheep Effect”: Extremity of judgments towards ingroup members as a function of group identification. European Journal of Social Psychology, 18, 1–16. doi:10.1002/ejsp.2420180102

[bibr119-1745691620902442] McLemore K. A. (2015). Experiences with misgendering: Identity misclassification of transgender spectrum individuals. Self and Identity, 14, 51–74. doi:10.1080/15298868.2014.950691

[bibr120-1745691620902442] Miller L. R. Grollman E. A. (2015). The social costs of gender-nonconformity for transgender adults: Implications for discrimination and health. Sociological Forum, 30, 809–831. doi:10.1111/socf.12193 27708501 PMC5044929

[bibr121-1745691620902442] Mohr J. J. Rochlen A. B. (1999). Measuring attitudes regarding bisexuality in lesbian, gay male, and heterosexual populations. Journal of Counseling Psychology, 46, 353–369. doi:10.1037/0022-0167.46.3.353

[bibr122-1745691620902442] Mol A. (1985). Wie Weet Wat een Vrouw Is. . . . Over de Verschillen en de Verhoudingen tussen de Wetenschappen [Who knows what a woman is. . . . about the differences and relationships between the sciences]. Tijdschrift voor Vrouwenstudies, 21, 10–22.

[bibr123-1745691620902442] Mol A. (2015). Who knows what a woman is . . . On the differences and the relations between the sciences. Medicine Anthropology Theory, 2, 57–75.

[bibr124-1745691620902442] Morgenroth T. Ryan M. K. (2018). Gender trouble in social psychology: How can Butler’s work inform experimental social psychologists’ conceptualization of gender? Frontiers in Psychology, 9, Article 1320. doi:10.3389/fpsyg.2018.01320 PMC6072877 30100895

[bibr125-1745691620902442] Morton T. A. Postmes T. (2009). When differences become essential: Minority essentialism in response to majority treatment. Personality and Social Psychology Bulletin, 35, 656–668. doi:10.1177/0146167208331254 19228599

[bibr126-1745691620902442] Mosher C. Levitt H. M. Manley E. (2006). Layers of leather: The identity formation of leathermen as a process of transforming meanings of masculinity. Journal of Homosexuality, 51, 93–123. doi:10.1300/J082v51n03_06 17135117

[bibr127-1745691620902442] Moss-Racusin C. A. Phelan J. E. Rudman L. A. (2010). When men break the gender rules: Status incongruity and backlash against modest men. Psychology of Men & Masculinity, 11, 140–152. doi:10.1037/a0018093

[bibr128-1745691620902442] Mulick P. S. Wright L. W. Jr. (2002). Examining the existence of biphobia in the heterosexual and homosexual populations. Journal of Bisexuality, 2(4), 45–64. doi:10.1300/J159v02n04_03

[bibr129-1745691620902442] Nagoshi C. T. Cloud J. R. Lindley L. M. Nagoshi J. L. Lothamer L. J. (2019). A test of the three-component model of gender-based prejudices: Homophobia and transphobia are affected by raters’ and targets’ assigned sex at birth. Sex Roles, 80, 137–146. doi:10.1007/s11199-018-0919-3

[bibr130-1745691620902442] Nagoshi J. L. Brzuzy S. I. Terrell H. K. (2012). Deconstructing the complex perceptions of gender roles, gender identity, and sexual orientation among transgender individuals. Feminism & Psychology, 22, 405–422. doi:10.1177/0959353512461929

[bibr131-1745691620902442] Napier J. L. Van der Toorn J. Vial A. C. (2019, July). The personal is political: Sexual stigma and the desire for gender-complementary relationships among gay men. Paper presented at the annual meeting of the International Society for Political Psychology, Lisbon, Portugal.

[bibr132-1745691620902442] National Institute of Mental Health. (2019, April). Suicide. Retrieved from https://www.nimh.nih.gov/health/statistics/suicide.shtml

[bibr133-1745691620902442] Netchaeva E. Kouchaki M. Sheppard L. D. (2015). A man’s (precarious) place: Men’s experienced threat and self-assertive reactions to female superiors. Personality and Social Psychology Bulletin, 41, 1247–1259. doi:10.1177/0146167215593491 26162611

[bibr134-1745691620902442] Newton E. (1968). The drag queens: A study in urban anthropology (Doctoral dissertation). University of Chicago, IL.

[bibr135-1745691620902442] Norton A. T. Herek G. M. (2013). Heterosexuals’ attitudes toward transgender people: Findings from a national probability sample of U.S. adults. Sex Roles, 68, 738–753. doi:10.1007/s11199-011-0110-6

[bibr136-1745691620902442] Olson K. R. Gülgöz S. (2018). Early findings from the TransYouth project: Gender development in transgender children. Child Development Perspectives, 12, 93–97. doi:10.1111/cdep.12268

[bibr137-1745691620902442] Olson K. R. Key A. C. Eaton N. R. (2015). Gender cognition in transgender children. Psychological Science, 26, 467–474. doi:10.1177/0956797614568156 25749700

[bibr138-1745691620902442] Outten H. R. Lee T. Lawrence M. E. (2019). Heterosexual women’s support for trans-inclusive bathroom legislation depends on the degree to which they perceive trans women as a threat. Group Processes & Intergroup Relations, 22(8), 1094–1108. doi:10.1177/1368430218812660

[bibr139-1745691620902442] Panesis C. P. Levitt H. M. Bridges S. K. (2014). The sexuality within butch and femme sexual minority women (Honors thesis). University of Massachusetts, Boston, MA.

[bibr140-1745691620902442] Parveen N. (2019, March 23). Activist warning of ‘war on morality’ wades into LGBT lessons row. The Guardian. Retrieved from https://www.theguardian.com

[bibr141-1745691620902442] Prince V. (2005). Sex vs. gender. International Journal of Transgenderism, 8(4), 29–32. doi:10.1300/J485v08n04_05

[bibr142-1745691620902442] Purdie-Vaughns V. Eibach R. P. (2008). Intersectional invisibility: The distinctive advantages and disadvantages of multiple subordinate-group identities. Sex Roles, 59, 377–391. doi:10.1007/s11199-008-9424-4

[bibr143-1745691620902442] Reich C. G. Taylor M. E. McCarthy M. M. (2009). Differential effects of chronic unpredictable stress on hippocampal CB1 receptors in male and female rats. Behavioural Brain Research, 203, 264–269. doi:10.1016/j.bbr.2009.05.013 19460405 PMC2747651

[bibr144-1745691620902442] Rhodes M. Gelman S. A. (2009). A developmental examination of the conceptual structure of animal, artifact, and human social categories across two cultural contexts. Cognitive Psychology, 59, 244–274. doi:10.1016/j.cogpsych.2009.05.001 19524886 PMC2770000

[bibr145-1745691620902442] Rivas J. (2015). Half of young people believe gender isn’t limited to male and female. Retrieved from http://fusion.kinja.com/half-of-youngpeople-believe-gender-isnt-limited-to-mal-1793844971

[bibr146-1745691620902442] Roberts S. O. Ho A. K. Rhodes M. Gelman S. A. (2017). Making boundaries great again: Essentialism and support for boundary-enhancing initiatives. Personality and Social Psychology Bulletin, 43, 1643–1658. doi:10.1177/0146167217724801 28914160

[bibr147-1745691620902442] Rothblum E. D. Balsam K. F. Wickham R. E. (2018). Butch, femme, and androgynous gender identities within female same sex couples: An actor-partner analysis. Psychology of Sexual Orientation and Gender Diversity, 5, 72–81. doi:10.1037/sgd0000258

[bibr148-1745691620902442] Rudman L. A. Moss-Racusin C. A. Glick P. Phelan J. E. (2012). Reactions to vanguards. In Advances in experimental social psychology (Vol. 45, pp. 167–227). San Diego, CA: Academic Press. doi:10.1016/B978-0-12-394286-9.00004-4

[bibr149-1745691620902442] Rudman L. A. Moss-Racusin C. A. Phelan J. E. Nauts S. (2012). Status incongruity and backlash effects: Defending the gender hierarchy motivates prejudice against female leaders. Journal of Experimental Social Psychology, 48, 165–179. doi:10.1016/j.jesp.2011.10.008

[bibr150-1745691620902442] Sax L. (2002). How common is intersex? A response to Anne Fausto-Sterling. Journal of Sex Research, 39, 174–178. doi:10.1080/00224490209552139 12476264

[bibr151-1745691620902442] Schmitt M. T. Branscombe N. R. (2001). The good, the bad, and the manly: Threats to one’s prototypicality and evaluations of fellow in-group members. Journal of Experimental Social Psychology, 37, 510–517. doi:10.1006/jesp.2001.1476

[bibr152-1745691620902442] Schmitt M. T. Lehmiller J. J. Walsh A. L. (2007). The role of heterosexual identity threat in differential support for same-sex ‘civil unions’ versus ‘marriages.’ Group Processes & Intergroup Relations, 10, 443–455. doi:10.1177/1368430207081534

[bibr153-1745691620902442] Schudson Z. C. Beischel W. J. van Anders S. M. (2019). Individual variation in gender/sex category definitions. Psychology of Sexual Orientation and Gender Diversity, 6, 448–460. doi:10.1037/sgd0000346

[bibr154-1745691620902442] Schudson Z. C. Manley M. H. Diamond L. M. van Anders S. M . (2018). Heterogeneity in gender/sex sexualities: An exploration of gendered physical and psychological traits in attractions to women and men. The Journal of Sex Research, 55(8), 1077–1085. 29190144 10.1080/00224499.2017.1402290

[bibr155-1745691620902442] Seelman K. L. (2014). Transgender individuals’ access to college housing and bathrooms: Findings from the National Transgender Discrimination Survey. Journal of Gay & Lesbian Social Services, 26, 186–206. doi:10.1080/10538720.2014.891091

[bibr156-1745691620902442] Sender K. (2004). Neither fish nor fowl: Feminism, desire, and the lesbian consumer market. The Communication Review, 7, 407–432. doi:10.1080/10714420490886989

[bibr157-1745691620902442] Sesko A. K. Biernat M. (2010). Prototypes of race and gender: The invisibility of Black women. Journal of Experimental Social Psychology, 46, 356–360. doi:10.1016/j.jesp.2009.10.016

[bibr158-1745691620902442] Sharp J. (2018, August 27). ‘Protect our children’: Baptist ministers, supporters speak out against drag queen story hour. Alabama Media Group. Retrieved from https://www.al.com/news/2018/08/protect_our_children_baptist_m.html

[bibr159-1745691620902442] Sherkat D. E. Powell-Williams M. Maddox G. De Vries K. M. (2011). Religion, politics, and support for same-sex marriage in the United States, 1988–2008. Social Science Research, 40, 167–180. doi:10.1016/j.ssresearch.2010.08.009

[bibr160-1745691620902442] Slawson N. (2018, October 20). How possible changes to the Gender Recognition Act prompted a toxic debate. HuffPost. Retrieved from https://www.huffingtonpost.co.uk

[bibr161-1745691620902442] Smith S. G. Zhang X. Basile K. C. Merrick M. T. Wang J. Kresnow M. Chen J. (2018). The National Intimate Partner and Sexual Violence Survey (NISVS): 2015 data brief—updated release. Atlanta, GA: National Center for Injury Prevention and Control, Centers for Disease Control and Prevention. Retrieved from https://www.cdc.gov/violenceprevention/pdf/2015data-brief508.pdf

[bibr162-1745691620902442] Steinmetz K. (2016, June 14). From horse people to Hillary Clinton: A history of women wearing pants. Time. Retrieved from http://time.com

[bibr163-1745691620902442] Stephan W. G. Ybarra O. Morrison K. R. (2009). Intergroup threat theory. In Nelson T. D. (Ed.), Handbook of prejudice, stereotyping, and discrimination (pp. 43–59). New York, NY: Psychology Press.

[bibr164-1745691620902442] Stern C. Rule N. O. (2018). Physical androgyny and categorization difficulty shape political conservatives’ attitudes toward transgender people. Social Psychological & Personality Science, 9, 24–31.

[bibr165-1745691620902442] Stock K. (2018, October 1). Why self-identification should not legally make you a woman. The Conversation. Retrieved from https://theconversation.com/why-self-identification-should-not-legally-make-you-a-woman-103372

[bibr166-1745691620902442] Stones R. J. (2017). Which gender is more concerned about transgender women in female bathrooms? Gender Issues, 34, 275–291. doi:10.1007/s12147-016-9181-6

[bibr167-1745691620902442] Tajfel H. Turner J. C. (1979). An integrative theory of intergroup conflict. In Austin W. G. Worchel S. (Eds.), The social psychology of intergroup relations (pp. 33–37). Monterey, CA: Brooks/Cole.

[bibr168-1745691620902442] Tebbe E. N. Moradi B. (2012). Anti-transgender prejudice: A structural equation model of associated constructs. Journal of Counseling Psychology, 59, 251–261. doi:10.1037/a0026990 22329343

[bibr169-1745691620902442] Trotta D. (2016, April 21). Exclusive: Women, young more open on transgender issue in U.S. – Reuters/Ipsos poll. Reuters. Retrieved from http://www.reuters.com/article/us-usa-lgbt-poll-idUSKCN0XI11M

[bibr170-1745691620902442] Tulchinsky D. Hobel C. J. Yeager E. Marshall J. R. (1972). Plasma estrone, estradiol, estriol, progesterone, and 17-hydroxyprogesterone in human pregnancy. I. Normal pregnancy. American Journal of Obstetrics & Gynecology, 112, 1095–1100. doi:10.1016/0002-9378(72)90185-8 5025870

[bibr171-1745691620902442] Turner J. C. Hogg M. A. Oakes P. J. Reicher S. D. Wetherell M. S. (1987). Rediscovering the social group: A self-categorization theory. Oxford, England: Blackwell.

[bibr172-1745691620902442] van Anders S. M. (2013). Beyond masculinity: Testosterone, gender/sex, and human social behavior in a comparative context. Frontiers in Neuroendocrinology, 34, 198–210. doi:10.1016/j.yfrne.2013.07.001 23867694

[bibr173-1745691620902442] van Anders S. M. (2015). Beyond sexual orientation: Integrating gender/sex and diverse sexualities via sexual configurations theory. Archives of Sexual Behavior, 44, 1177–1213. doi:10.1007/s10508-015-0490-8 25772652

[bibr174-1745691620902442] van Anders S. M. Steiger J. Goldey K. L. (2015). Effects of gendered behavior on testosterone in women and men. Proceedings of the National Academy of Sciences, USA, 112, 13805–13810. doi:10.1073/pnas.1509591112 PMC4653185 26504229

[bibr175-1745691620902442] Vandello J. A. Bosson J. K. Cohen D. Burnaford R. M. Weaver J. R. (2008). Precarious manhood. Journal of Personality and Social Psychology, 95, 1325–1339. doi:10.1037/a0012453 19025286

[bibr176-1745691620902442] van der Toorn J. Tyler T. R. Jost J. T. (2011). More than fair: Outcome dependence, system justification, and the perceived legitimacy of authority. Journal of Experimental Social Psychology, 47, 127–138. doi:10.1016/j.jesp.2010.09.003

[bibr177-1745691620902442] Vaughn A. A. Teeters S. A. Sadler M. S. Cronan S. B. (2017). Stereotypes, emotions, and behaviors toward lesbians, gay men, bisexual women, and bisexual men. Journal of Homosexuality, 64, 1890–1911. doi:10.1080/00918369.2016.1273718 27982743

[bibr178-1745691620902442] Viki G. T. Abrams D. (2002). But she was unfaithful: Benevolent sexism and reactions to rape victims who violate traditional gender role expectations. Sex Roles, 47, 289–293. doi:10.1023/A:1021342912248

[bibr179-1745691620902442] Wagoner J. A. Belavadi S. Jung J. (2017). Social identity uncertainty: Conceptualization, measurement, and construct validity. Self and Identity, 16, 505–530. doi:10.1080/15298868.2016.1275762

[bibr180-1745691620902442] Warriner K. Nagoshi C. T. Nagoshi J. L. (2013). Correlates of homophobia, transphobia, and internalized homophobia in gay or lesbian and heterosexual samples. Journal of Homosexuality, 60, 1297–1314. doi:10.1080/00918369.2013.806177 23952924

[bibr181-1745691620902442] Wason P. C. (1960). On the failure to eliminate hypotheses in a conceptual task. Quarterly Journal of Experimental Psychology, 12, 129–140.

[bibr182-1745691620902442] Weber R. Crocker J. (1983). Cognitive processes in the revision of stereotypic beliefs. Journal of Personality and Social Psychology, 45, 961–977. doi:10.1037/0022-3514.45.5.961

[bibr183-1745691620902442] Williams C. (2016). Radical inclusion: Recounting the trans inclusive history of radical feminism. Transgender Studies Quarterly, 3, 254–258. doi:10.1215/23289252-3334463

[bibr184-1745691620902442] Wilton L. S. Bell A. N. Carpinella C. M. Young D. M. Meyers C. Clapham R. (2019). Lay theories of gender influence support for women and transgender people’s legal rights. Social Psychological & Personality Science, 10, 883–894. doi:10.1177/1948550618803608

[bibr185-1745691620902442] Wood W. Eagly A. H. (2015). Two traditions of research on gender identity. Sex Roles, 73, 461–473. doi:10.1007/s11199-015-0480-2

[bibr186-1745691620902442] World Health Organization. (2016). Gender identity disorders. In International Classification of Diseases 10th revision. Geneva, Switzerland: Author. Retrieved from https://icd.who.int/browse10/2016/en#/F60-F69

[bibr187-1745691620902442] Yeung A. W. Y. Kay A. C. Peach J. M. (2014). Anti-feminist backlash: The role of system justification in the rejection of feminism. Group Processes & Intergroup Relations, 17, 474–484. doi:10.1177/1368430213514121

[bibr188-1745691620902442] Yoder J. (2003). Women and gender: Transforming psychology (2nd ed.). Upper Saddle River, NJ: Prentice Hall.

[bibr189-1745691620902442] Zingora T. Graf S. (2019). Marry who you love: Intergroup contact with gay people and another stigmatized minority is related to voting on the restriction of gay rights through threat. Journal of Applied Social Psychology, 49, 684–703. doi:10.1111/jasp.12627

